# Diagnostic test evaluation methodology: A systematic review of methods employed to evaluate diagnostic tests in the absence of gold standard – An update

**DOI:** 10.1371/journal.pone.0223832

**Published:** 2019-10-11

**Authors:** Chinyereugo M. Umemneku Chikere, Kevin Wilson, Sara Graziadio, Luke Vale, A. Joy Allen

**Affiliations:** 1 Institute of Health & Society, Faculty of Medical Sciences Newcastle University, Newcastle upon Tyne, England, United Kingdom; 2 School of Mathematics, Statistics and Physics, Newcastle University, Newcastle upon Tyne, England, United Kingdom; 3 National Institute for Health Research, Newcastle In Vitro Diagnostics Co-operative, Newcastle upon Tyne Hospitals National Health Services Foundation Trust, Newcastle upon Tyne, England, United Kingdom; 4 National Institute for Health Research, Newcastle In Vitro Diagnostics Co-operative, Newcastle University, Newcastle upon Tyne, England, United Kingdom; Universita degli Studi di Firenze, ITALY

## Abstract

**Objective:**

To systematically review methods developed and employed to evaluate the diagnostic accuracy of medical test when there is a missing or no gold standard.

**Study design and settings:**

Articles that proposed or applied any methods to evaluate the diagnostic accuracy of medical test(s) in the absence of gold standard were reviewed. The protocol for this review was registered in PROSPERO (CRD42018089349).

**Results:**

Identified methods were classified into four main groups: methods employed when there is a missing gold standard; correction methods (which make adjustment for an imperfect reference standard with known diagnostic accuracy measures); methods employed to evaluate a medical test using multiple imperfect reference standards; and other methods, like agreement studies, and a mixed group of alternative study designs. Fifty-one statistical methods were identified from the review that were developed to evaluate medical test(s) when the true disease status of some participants is unverified with the gold standard. Seven correction methods were identified and four methods were identified to evaluate medical test(s) using multiple imperfect reference standards. Flow-diagrams were developed to guide the selection of appropriate methods.

**Conclusion:**

Various methods have been proposed to evaluate medical test(s) in the absence of a gold standard for some or all participants in a diagnostic accuracy study. These methods depend on the availability of the gold standard, its’ application to the participants in the study and the availability of alternative reference standard(s). The clinical application of some of these methods, especially methods developed when there is missing gold standard is however limited. This may be due to the complexity of these methods and/or a disconnection between the fields of expertise of those who develop (e.g. mathematicians) and those who employ the methods (e.g. clinical researchers). This review aims to help close this gap with our classification and guidance tools.

## Introduction

**Fig 1:**
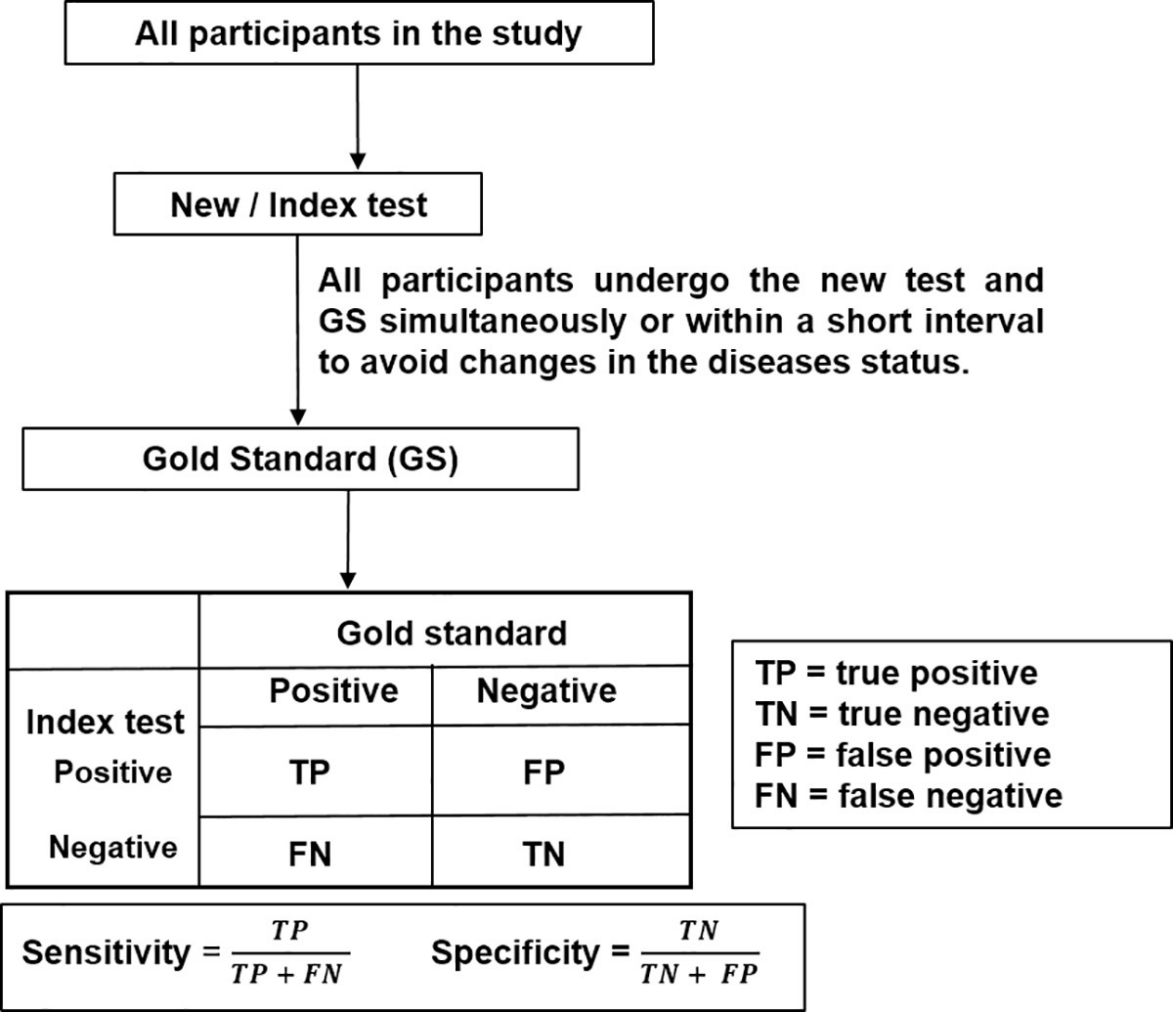
Classical method of evaluating the diagnostic accuracy of a medical test with binary test result and dichotomized disease status.

Before a new medical test can be introduced into clinical practice, it should be evaluated for analytical validity (does the test work in the laboratory?), clinical validity (does the test work in the patient population of interest?) and clinical utility (is the test useful–can it lead to improvement in health outcomes?) [1, 2]. Clinical validity studies, also called diagnostic accuracy studies, evaluate the test’s accuracy in discriminating between patients with or without the target condition (disease) [3]. The characteristics of the test (e.g. sensitivity and specificity) may inform what role the index test (the new test under evaluation) plays in the diagnostic pathway; is it a triage, add-on or replacement test? [4] Sensitivity (the proportion of participants correctly identified by the index test as having the target condition e.g. those with the disease) and specificity (the proportion of participants correctly identified by the index as not having the target condition) [5–7] are basic measures of the diagnostic accuracy of a test. Other common measures are predictive values, likelihood values, overall accuracy [8, 9], receiver operating characteristic (ROC) curve, area under the ROC curve (AUROC) [10], ROC surface, and volume under the ROC surface (VUS) [11–13]. These measures are obtained by comparing the index test results with the results of the best currently available test for diagnosing the same target condition in the same participants; both tests are supposedly applied to all participants of the study [14]. The test employed as the benchmark to evaluate the index test is called the reference standard [15]. The reference standard could be a gold standard (GS), with sensitivity and specificity equal to 100%. This means that the gold standard perfectly discriminates between participants with or without the target conditions and provides unbiased estimates of the diagnostic accuracy measure of the index test as describe in Fig 1. The term “bias” in this review is defined as the difference between the estimated value and the true value of the parameter of interest [16].

It is also expected that when evaluating the diagnostic accuracy of a medical test, the participants undertake both the index and reference tests within a short time-period if not simultaneously. This is to avoid biases caused by changes in their true disease status, which can also affect the diagnostic accuracy of the index test.

In addition to the common aforementioned diagnostic accuracy measures, there are other ways to evaluate the test performance of an index test. These include studies of agreement or concordance [17] between the index test and the reference standard and test positivity (or negativity) rate; that is the proportion of diagnostic tests that are positive (or negative) to the target condition [18].

In practice, there are deviations from the classical method (Fig 1). These deviations are:

1. Scenarios where the gold standard is not applied to all participants in the study (i.e. there is a missing gold standard) because it is expensive, or invasive, or patients do not consent to it, or the clinicians decided not to give the gold test to some patients for medical reasons [19, 20]. Evaluating the new test using data only from participants whose disease status was confirmed with the gold standard can produce work-up or verification bias [21].
2. Scenarios where the reference standard is not a gold standard (i.e. it is an imperfect reference standard) because it has a misclassification error or because there is no generally accepted reference standard for the target condition. Using an imperfect reference standard produces reference standard bias [22, 23].

Several methods have been developed and used to evaluate the test performance of a medical test in these two scenarios.

Reviews of some of these methods have been undertaken previously. The reviews by Zhou [24], Alonzo [25] and the report by Naaktgeboren et al [26] focused on methods when the gold standard or reference standard is not applied to all participants in the study; Van Smeden et al [27] and Collins and Huynh [28] focused on the latent class models (LCMs); and Hui and Zhou [29], Trikalinos and Balion [30] and Enoe et al [31] focused on methods employed when the reference standard is imperfect. Zaki et al [32] focused on the agreement between medical tests whose results are reported as a continuous response. Branscum et al [33] focused on Bayesian approaches; and the reviews by Walsh [23], Rutjes et al [14] and Reitsma et al [34] focused around methods for evaluating diagnostic tests when there is a missing or imperfect reference standard.

The existing comprehensive reviews on this topic were published about 11 years ago [14, 34]; knowledge, ideas, and research in this field has evolved significantly since then. Several new methods have been proposed and some existing methods have been modified. It is also possible that some previously identified methods may now be obsolete. Therefore, one of the aims of this systematic review is to review new and existing methods employed to evaluate the test performance of medical test(s) in the absence of gold standard for all or some of the participants in the study. It also aims to provide easy to use tools (flow-diagrams) for the selection of methods to consider when evaluating medical tests when sub-sample of the participants do not undergo the gold standard. The review builds upon the earlier reviews by Rutjes et al and Reitsma et al [14, 34]. This review sought to identify methods developed to evaluate a medical test with continuous results in the presence of verification bias and when the diagnostic outcome (disease status) is classified into three or more groups (e.g. diseased, intermediate and non-diseased). This is a gap identified in the review conducted by Alonzo [25] in 2014.

The subsequent sections discuss the method employed to undertake the review, the results, the discussion of the findings and guidance to researchers involved in test accuracy studies.

## Methodology

A protocol for this systematic review was developed, peer-reviewed and registered on PROSPERO (CRD42018089349).

### Eligibility criteria

The review includes methodological articles (that is papers that proposed or developed a method) and application articles (that is papers where any of the proposed methods) were applied.

#### Inclusion

- Articles published in English language in a peer-reviewed journal.
- Articles that focus on evaluating the diagnostic accuracy of new (index) test when there is a missing gold standard, no gold standard or imperfect reference standard.

#### Exclusion

- Articles that assumed that the reference standard was a gold standard and the gold standard was applied to all participants in the study.
- Books, dissertations, thesis, conference abstracts, and articles not published in a peer reviewed journal.
- Systematic reviews and meta-analyses of the diagnostic accuracy of medical test(s) for a target condition (disease) in the absence of gold standard for some or all of the participants. However, individual articles included in these reviews that met the inclusion criteria were included.

### Search strategies and selection of articles

The PRISMA statement [35] was used as a guideline when conducting this systematic review. The PRISMA checklist for this review, S1 Checklist, is included as one of the supplementary materials. The following bibliographic databases were searched: EMBASE, MEDLINE, SCOPUS, WILEY online library (which includes Cochrane library, EBM), PSYCINFO, Web of Science, and CINAHL. The details of the search strategies at reported in the S1 Appendix. The search dates were from January 2005 –February 2019. This is because, this review is an update of a review by Rutjes et al and Reitsma et al whose searched up to 2005. However, original methodological articles that proposed and described a method to evaluate medical test(s) when there is a missing or no gold standard published before 2005 were also included in the review. These original articles were identified by "snowballing" [36] from the references of some articles. All articles obtained from the electronic databases were imported to Endnote X8.0.2. The selection of articles to be included in this review were done by three people (CU, AJA, and KW). The sifting process was in two-stages: by title and abstract and then by full text against the inclusion and exclusion criteria. Any discrepancies between reviewers were resolved in a group meeting.

### Data synthesis

A data collection form was developed for this review (S1 Data), which was piloted on seven studies and remodified to fit the purpose of this review. Information extracted from the included articles were synthesized narratively.

## Results

**Fig 2:**
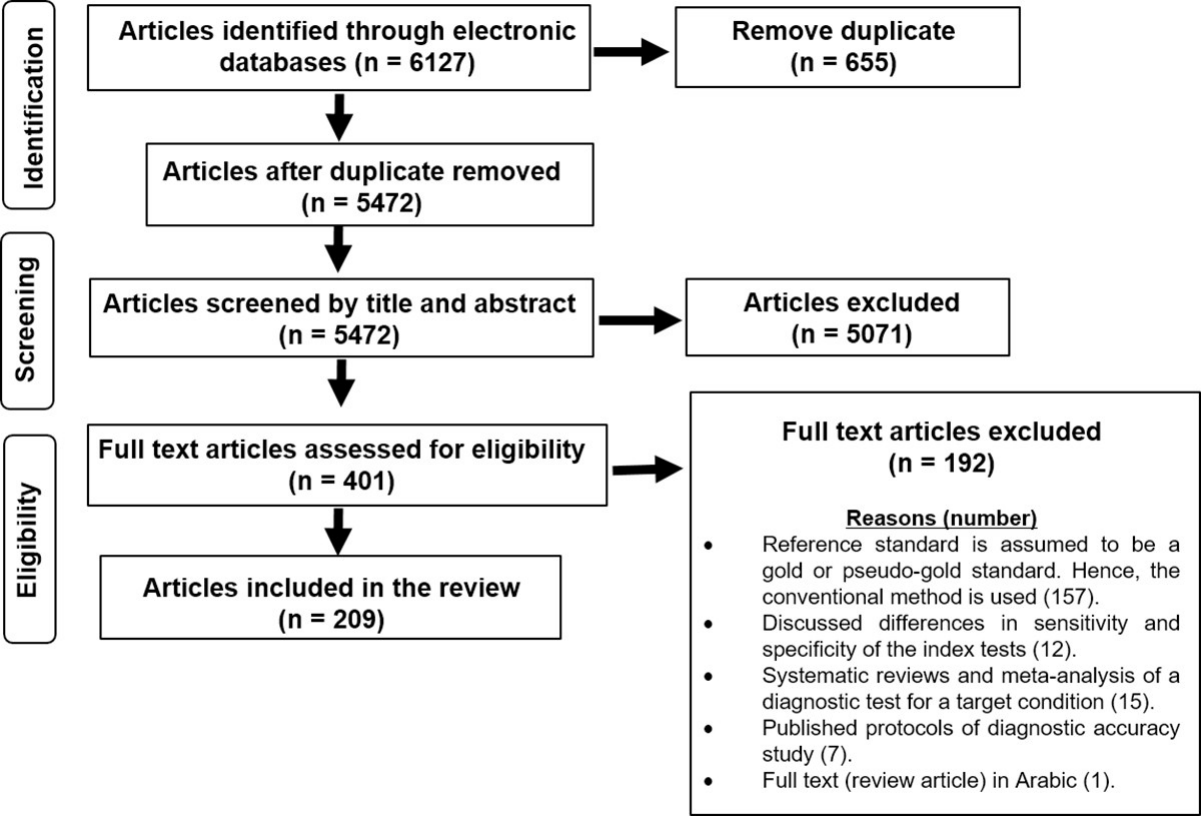
PRISMA flow-diagram of articles selected and included in the systematic review.

A total of 6127 articles were identified; 5472 articles were left after removing the duplicated articles; 5071 articles were excluded after sifting by title and abstract; 401 articles went forward to full text assessment; and a total of 209 articles were included in the review. The search and selection procedure are depicted using the PRISMA [35] flow-diagram (Fig 2).

The articles included in this review used a wide variety of different study designs, like cross-sectional studies, retrospective studies, cohort studies, prospective studies and simulation studies.

The identified methods were categorized into four groups based on the availability and/or application of the gold standard to the participants in the study. These group are:

- Group 1: Methods employed when there is a missing gold standard.
- Group 2: Correction methods which adjust for using an imperfect reference standard whose diagnostic accuracy is known.
- Group 3: Methods employed when using multiple imperfect reference standards.
- Group 4: ***“other methods”***. This group includes methods like study of agreement, test positivity rate, and considering alternative study design like validation.

Methods in groups 2, 3 and 4 are employed when there is no gold standard to evaluate the diagnostic accuracy of the index test; while methods in group 1 are employed when there is a gold standard to evaluate the diagnostic accuracy of the index test(s). However, the gold standard is applied to only a sub-sample of the participants.

A summary of all methods identified in the review, their key references and the clinical applications of these methods are reported on Table 1.

**Table 1:** Summary of classification of methods employed when there is missing or no gold standard.

Main Classification	Main Characteristics	Key references	Clinical Application
**Group 1**: **Method employed when there is missing gold standard:** • Imputation and bias-correction methods • Differential verification	The true disease status is verified with the gold standard only in a subsample of the study participants. The methods are grouped into ***imputation and bias-correction methods*** (**Fig 3**: Imputation and bias–correction methods in binary diagnostic outcomes. and **Fig** 4: Imputation and bias–correction methods in three- classes diagnostic outcomes where ROC surface and VUS are estimated.**)** and ***differential verification*** approach.	***Imputation and bias correction methods*** [10], [11], [13], [21], [37–81] ***Differential verification*** [82–84]	***Imputation & Bias-correction methods*** [85–89] ***Differential verification*** [90]
**Group 2**: **Correction methods**	The reference standard is imperfect. However, there is available information about the sensitivity and specificity of the reference standard which is used to correct or adjust the estimated sensitivity and specificity of the index test.	***Correction methods*** [91–96]	***Correction methods*** [97–99]
**Group 3: Methods employed when using multiple imperfect reference standards or tests.** • Discrepancy analysis • Latent class analysis • Composite reference standard (CRS) • Expert or panel or consensus diagnosis	A gold standard that diagnoses a target condition or accurate information on the diagnostic accuracy of an imperfect reference standard that diagnoses same condition may not be available. Thus, multiple imperfect tests may be employed to evaluate the index test. Methods in this group include discrepancy analysis, latent class analysis, composite reference standard, and panel or consensus diagnosis.	***Discrepancy analysis*** [100], [101] ***Latent class analysis*** ***Frequentist LCA***: [29],[102–112] ***Bayesian LCA***: [33], [113–119] ***ROC (NGS)***: [120–130] **Composite reference standard** [131–134] **Panel or consensus diagnosis** [135]	***Discrepancy analysis*** [136–139] ***Latent class analysis*** ***Frequentist LCA***: [140–152] ***Bayesian LCA***: [153–174] ***ROC (NGS)*:** [175, 176] ***CRS*:** [20, 177–184] ***Consensus diagnosis***: [185–189]
**Group 4**: **Other methods** • Considering an alternative study design like a validation study • Study of agreement • Test positivity rate	***Analytic validation*** of a medical test is the process of verifying the test based on what it is designed to do. Experimental or case-control are common designs for these studies. ***Studies of agreement*** aim to investigate the concordance between two or more tests (probably an index test and a reference standard). ***Test positivity rate***: is the proportion of participants who have positive results on a test. This approach was used by Van Dyck et al [18] to reduce the number of tests subjected to further evaluation.	***Validation*** [190, 191] ***Study of agreement***: [32], [192] ***Test positivity rate***: [18]	***Validation***: [193, 194] ***Study of agreement***: [165, 195–199] ***Test positivity rate*** [18, 192]

LCA: latent class analysis; CRS is composite reference standard. ROC is receiver operating characteristics; NGS is no gold standard

### Methods employed when gold standard is missing

**Fig 3:**
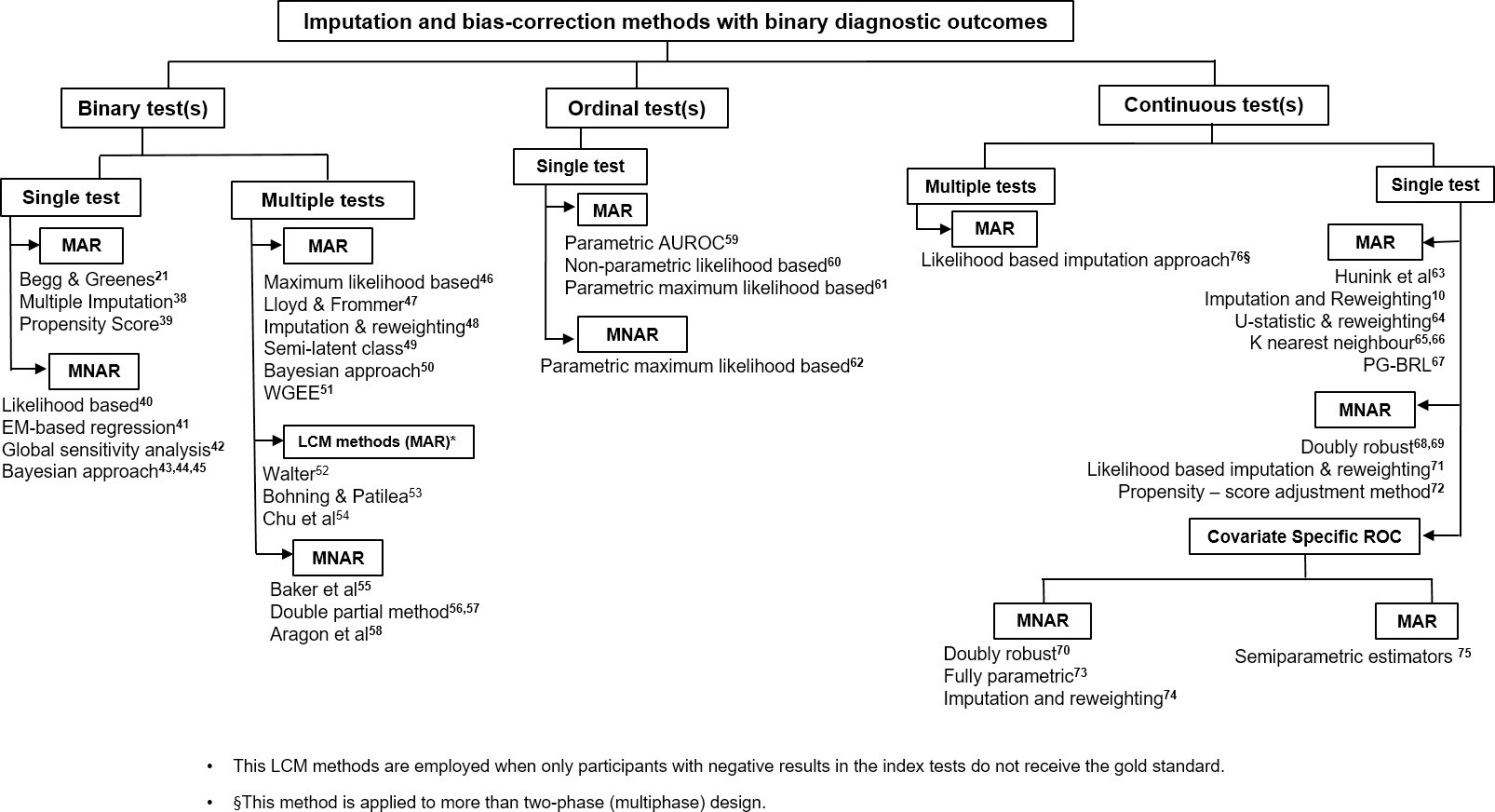
Imputation and bias–correction methods in binary diagnostic outcomes.

**Fig 4:**
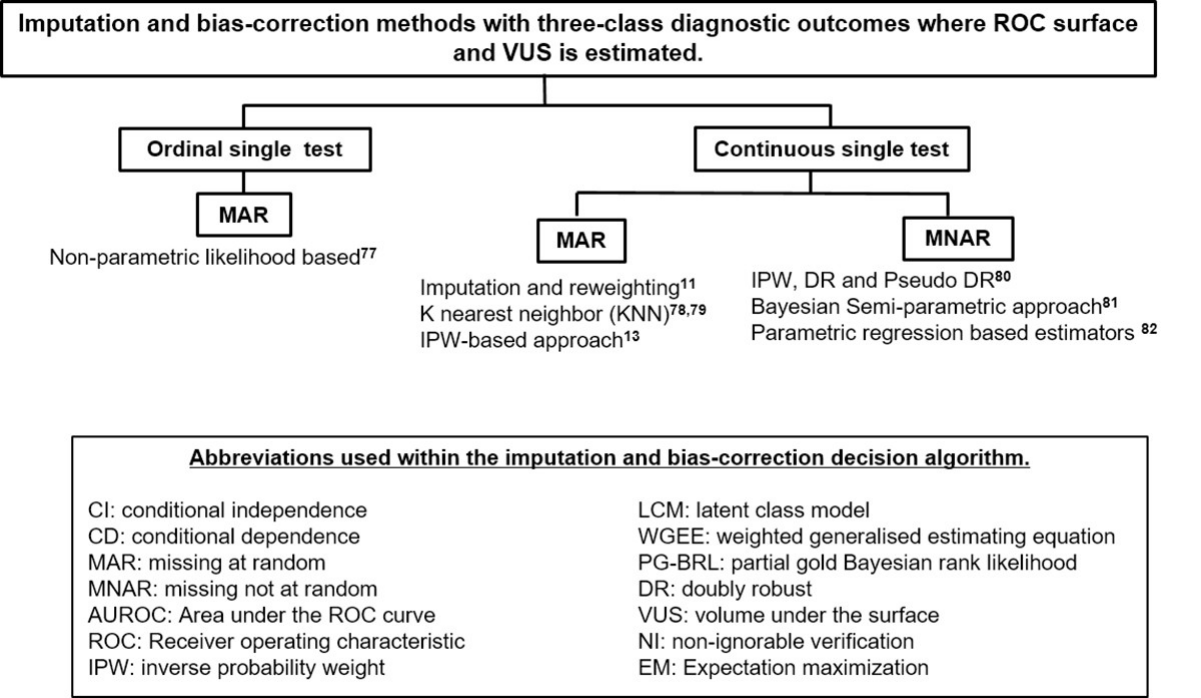
Imputation and bias–correction methods in three- classes diagnostic outcomes where ROC and VUS is estimated.

Fifty-one statistical methods were identified from the review that were developed to evaluate the diagnostic accuracy of index test(s) when the true disease status of some participants is not verified with the gold standard. These methods are divided into two subgroups:

- **Imputation and bias-correction methods**: This includes methods to correct for verification bias while the disease-status of the unverified participants are left unverified. Forty-eight statistical methods were identified in this group. These methods are further classified based on the result of the index test (binary, ordinal or continuous), the number of index tests evaluated (single or multiple), the assumptions made about verification (ignorable or missing at random–MAR) or non-ignorable or missing not at random–MNAR), and the classification of the diagnostic outcomes (disease-status). The identified methods in this subgroup are displayed Figs 3 and 4.

- **Differential verification approach**: Participants whose disease status was not verified with the gold standard could undergo another reference standard (that is imperfect or less invasive than the gold standard [84]) to ascertain their disease status. This is known as ***differential verification*** [200]. Differential verification has been explored Alonzo et al, De Groot et al and Naaktgeboren et al [200–202]. They discussed the bias associated with differential verification, and how results using this approach could be presented. There are three identified statistical methods in this group. They are: a Bayesian latent class approach proposed by De Groot et al [82], a Bayesian method proposed by Lu et al [203] and a ROC approach proposed by Glueck et al [16]. These three methods aim to simultaneously adjust for differential verification bias and reference standard bias that arises from using an alternative reference standard (i.e. imperfect reference standard) for participants whose true disease status was not verified with the gold standard.

### Correction methods

This group includes algebraic methods developed to correct the estimated sensitivity and specificity of the index test when the sensitivity and specificity of the imperfect reference standard is known. There are seven statistical methods in this group described in five different articles [91–95]. The methods by Emerson et al [95] does not estimate a single value for sensitivity or specificity, unlike the other correction methods [91–94] but produces an upper bound value and a lower bound value for the sensitivity and specificity of the index test. These bounded values are used to explain the uncertainty around the estimated sensitivity and specificity of the index test.

### Methods with multiple imperfect reference standards

A gold standard or accurate information about the diagnostic accuracy of the imperfect reference standard are often not available to evaluate the index test. In these situations, multiple imperfect reference standards can be employed to evaluate the index test. Methods in this group include:

- **Discrepancy analysis**: this compares the index test with an imperfect reference standard. Participants with discordant results undergo another imperfect test, called the resolver test, to ascertain their disease status. Discrepancy analysis is typically not recommended because it produces biased estimates [100, 204]. Modifications of this approach have been proposed [18, 101, 136]. In these, some of the participants with concordant responses (true positives and true negatives) are sampled to undertake the resolver test alongside participants with discordant responses (false negative–FN and false positive–FP). However, further research is needed to explore if these modified approaches are adequate to remove or reduce the potential bias.
- **Latent class analysis (LCA)**: The test performance of all the tests employed in the study are evaluated simultaneously using probabilistic models with the basic assumption that the disease status is latent or unobserved. There are frequentist LCAs and Bayesian LCAs. The frequentist LCAs use only the data from the participants in the study to estimate the diagnostic accuracy measures of the tests; while the Bayesian LCAs employ external information (e.g. expert opinion or estimates from previous research study) on the diagnostic accuracy measures of the tests evaluated in additional to the empirical data obtained from participants within the study. The LCAs assume that the tests (new test and reference standards) are either conditionally independent given the true disease status or the tests are conditionally dependent. To model the conditional dependence among the tests, various latent class model (LCM) with different dependence structure have been developed such as the Log-linear LCM [102], Probit LCM [103], extended log-linear and Probit LCM [108], Gaussian Random Effect LCM [105] and two-crossed random effect LCM [107] among others. However, some studies [205],[206] have shown that latent class models with different conditional dependence structures produce different estimates of sensitivities and specificities and each model still has a good fit. Thus, further research could be carried out to explore if each of the conditional dependence LCM are case specific.
- **Construct composite reference standard**: this method combines results from multiple imperfect tests (excluding the index test) with a predetermined rule to construct a reference standard that is used to evaluate the index test. By excluding the index test as part of the composite reference standard, incorporation bias can be avoided [131]. A novel method identified under the composite reference standard is the “dual composite reference standard” proposed by Tang et al [134].
- **Panel or consensus diagnosis**: this method uses the decision from a panel of experts to ascertain the disease status of each participant, which is then used to evaluate the index test.

### Other methods

This group includes methods that fit the inclusion criteria but could not be placed into the other three groups. They include study of agreement, test positivity rate and the use of an alternative study design such as analytical validation. Study of agreement and test positivity rate are best used as exploratory tools alongside other methods [152, 178] because they are not robust enough to assess the diagnostic ability of the medical test. Validation of a medical test cut across different disciplines in medicine such as psychology, laboratory or experimental medicine. With this approach, the medical test is assessed based on what it is designed to do [191]. Other designs include case-control designs (where the participants are known to have or not have the target condition) [207, 208], laboratory based studies or experimental studies which are undertaken with the aim to evaluate the analytical sensitivity and specificity of the index test [190, 209, 210].

## Guidance to researchers

**Fig 5:**
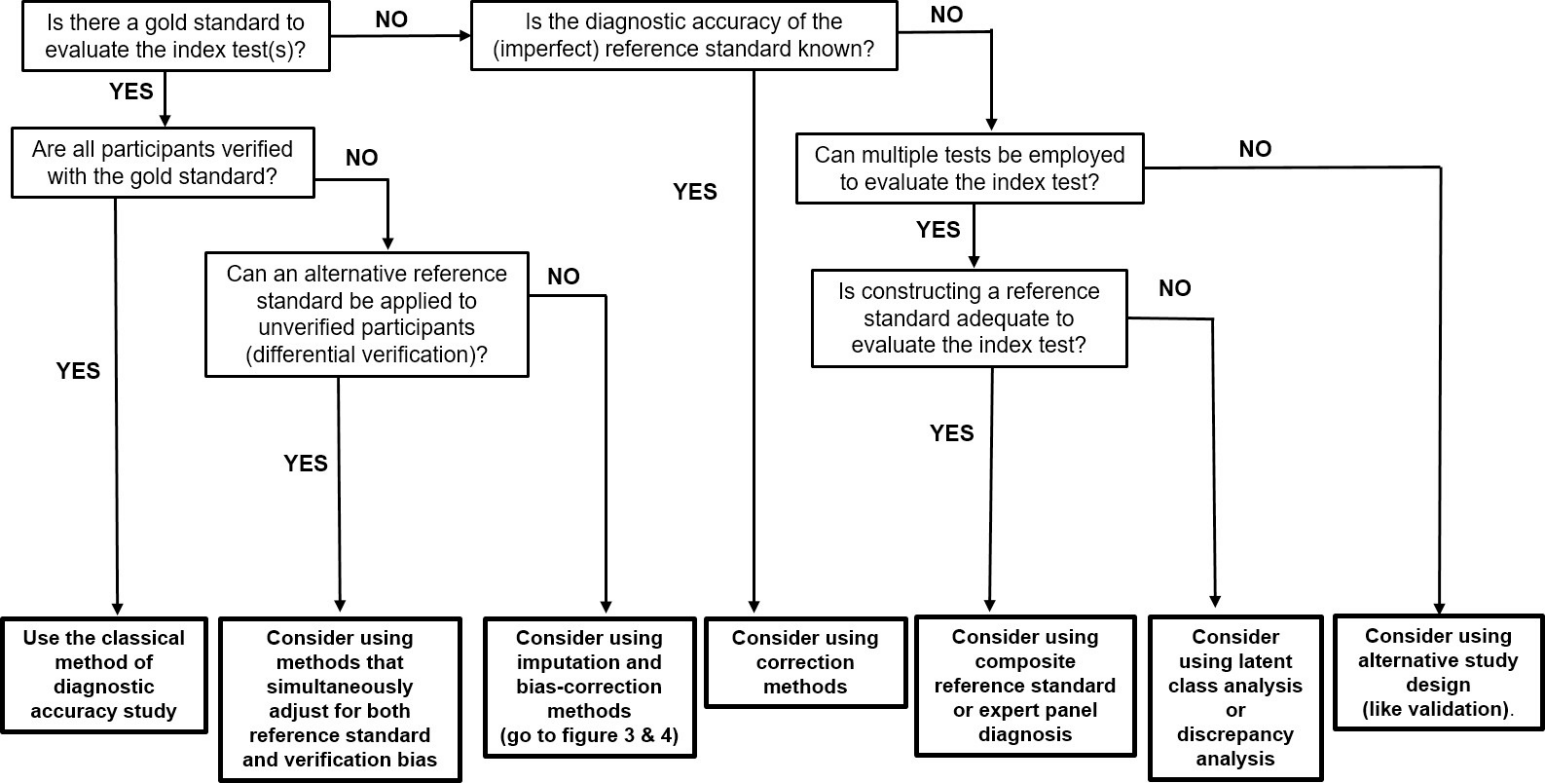
Guidance flowchart of methods employed to evaluate medical test in missing and no gold standard scenarios.

The guidance flowchart (Fig 5) is a modification and extension of the guidance for researchers flow-diagram developed by Reitsma et al [34].

Since, evaluating the accuracy measures of the index test is the focus of any diagnostic accuracy study, the flowchart starts with asking the first question “Is there a gold standard to evaluate the index test?” Following the responses from each question box (not bold); methods are suggested (bold boxes at the bottom of the flowchart) to guide clinical researchers, test evaluators, and researchers as to the different methods to consider.

Although, this review aims to provide up-to-date approaches that have been proposed or employed to evaluate the diagnostic accuracy of an index test in the absence of a gold standard for some or all of the participants in the accuracy study; some things researchers can consider when designing an accuracy study aside from the aim of their studies, are outlined in Box 1 ([26, 211–218]).

**Box 1: Suggestions when designing a diagnostic accuracy study.**

- **Design a protocol**: The protocol describes every step of the study. It states the problem and how it will be addressed.
- **Selection of participants from target population**: The target population determines the criteria for including participants in the study. Also, the population is important in selecting the appropriate setting for the study.
- **Selection of appropriate reference standard:** The reference standard should diagnose same target condition as the index test. The choice of reference standard (gold or non-gold) determines the methods to apply when evaluating the index test (see Fig 5).
- **Sample size**: Having adequate sample size is necessary to make precise inference from the statistical analysis that will be carried out. Studies that discuss the appropriate sample size to consider when planning test accuracy are [211–215].
- **Selection of accuracy measure to estimate**: The researchers need to decide which accuracy measures they wish to estimate, and this is often determined by the test’s response (binary or continuous).
- **Anticipate and eliminate possible bias**: multiple forms of bias may exist [26, 216–218]. Exploring how to avoid or adjust for these bias (if they are unavoidable) is important.
- **Validation of results**: Is validation of the results from the study on an independent sample feasible? Validation ensures an understanding of the reproducibility, strengths, and limitations of the study.

Some guidelines and tools have been developed to assist in designing, conducting and reporting diagnostic accuracy studies such as the STARD [219–223] guidelines, GATE [224] framework, QUADAS [225] tools; which can aid the design of a robust test accuracy study.

## Discussion

This review sought to identify and review new and existing methods employed to evaluate the diagnostic accuracy of a medical test in the absence of gold standard. The identified methods are classified into four main groups based on the availability and/or the application of the gold standard on the participants in the study. The four groups are: methods employed when only a sub-sample of the participants have their disease status verified with the gold standard (group 1); correction methods (group 2); methods using multiple imperfect reference standards (group 3) and other methods (group 4) such as study of agreement, test positivity rate and alternative study designs like validation.

In this review additional statistical methods have been identified that were not included in the earlier reviews on this topic by Reitsma et al [34] and Alonzo [25]. A list of all the methods identified in this review are presented in the supplementary material (S1 Supplementary Information). This includes a brief description of the methods and a discussion of their strengths and weaknesses and any identified case studies where the methods have been clinically applied. Only a small number of the methods we have identified have applied clinically and published [38, 63]. This may be due to the complexity of these methods (in terms of application and interpretation of results), and/or a disconnection between the fields of expertise of those who develop (e.g. mathematicians or statisticians) and those who employ the methods (e.g. clinical researchers). For example, the publication of such method in specialist statistical journals may not be readily accessible to clinical researchers designing the study. In order to close this gap, two flow-diagrams (Figs 3 and 4) were constructed in addition to the modified guidance flowchart, (Fig 5) as guidance tools to aid clinical researchers and test evaluators in the choice of methods to consider when evaluating medical test in the absence of gold standard. Also, an R package (*bcROCsurface*) and an interactive web application (Shiny app) that estimates the ROC surface and VUS in the presence of verification bias have been developed by To Duc [78] to help bridge the gap.

One of the issues not addressed in this current review was on methods that evaluate the differences in diagnostic accuracy of two or more tests in the presence of verification bias. Some published articles that consider this issue are Nofuentes and Del Castillo [226–230], Marin-Jimenez and Nofuentes [231], Harel and Zhou [232] and Zhou and Castelluccio [233]. This review also did not consider methods employed to estimate the time-variant sensitivity and specificity of diagnostic test in absence of a gold standard. This issue has recently been addressed by Wang et al [234].

In terms of the methodology, a limitation of this review is the exclusion of books, dissertations, thesis, conference abstract and articles not published in English language (such as the review by Masaebi et al [235] which was published in 2019), which could imply that there could still be some methods not identified by this review.

Regarding the methods identified in this review, further research could be carried to explore the different modification to the discrepancy analysis approaches to understand if these modifications reduce or remove the potential bias. In addition, further research is needed to determine if the different methods developed to evaluate an index test in the presence of verification bias are robust methods. Given the large numbers of statistical methods that have been developed especially to evaluate medical tests when there is a missing gold standard and the complexity of some of these methods; more interactive web application (e.g. Shiny package in R [236]) could be developed to implement these methods in addition to the Shiny app developed by To Duc [78] and Lim et al [237]. The development of such interactive web tools will expedite the clinical applications of these developed methods and help bridge the gap between the method developers and the clinical researchers or tests evaluators who are the end users of these methods.

## Conclusion

Various methods have been proposed and applied in the evaluation of medical tests when there is a missing gold standard result for some participants, or no gold standard at all. These methods depend on the availability of the gold standard, its application to all or subsample of participants in the study, the availability of alternative reference standard(s), and underlying assumption(s) made with respect to the index test(s) and / or participants in the study.

Knowing the appropriate method to employ when analysing the data from participants of a diagnostic accuracy studies in the absence of gold standard, help to make statistically robust inference on the accuracy of the index test. This, in addition to data on cost-effectiveness, utility and usability of the test will support clinicians, policy makers and stake holders to decide the adoption of the new test in practice or not.

## Supporting information

S1 Checklist PRISMA checklist. (DOC)

S1 Data Data extraction form. (DOCX)

S1 Appendix (DOCX)

S1 Supplementary Information (DOCX)
