# Supplementary material for: Diagnostic test evaluation methodology: A systematic review of methods employed to evaluate diagnostic tests in the absence of gold standard – An update
Source: PLoS One. 2019 Oct 11;14(10):e0223832. doi: 10.1371/journal.pone.0223832 (PMC6788703; doi:10.1371/journal.pone.0223832)
Supplement: S1 Data — (DOCX) [file pone.0223832.s002.docx]

**Data extraction Sheet**

| Date collected |  |
| --- | --- |
| Collector’s ID |  |
| Author(s) |  |
| Year |  |
| URL |  |
| Title |  |
| Source |  |
| Database |  |
| URL |  |
| Aim/objective of study |  |
| Type of study design |  |
| “No gold standard” type |  |
| Target Condition |  |
| Index test |  |
| Reference standard |  |
| Data |  |
| Time frame |  |
| Method |  |
| Notes | **Assumptions**  **Was it met?**  **Results** |
