# Supplementary material for: Diagnostic test evaluation methodology: A systematic review of methods employed to evaluate diagnostic tests in the absence of gold standard – An update
Source: PLoS One. 2019 Oct 11;14(10):e0223832. doi: 10.1371/journal.pone.0223832 (PMC6788703; doi:10.1371/journal.pone.0223832)
Supplement: S1 Appendix — (DOCX) [file pone.0223832.s003.docx]

# **Appendix:**

The detail of the search strategy using the SCOPUS database is:

( TITLE-ABS-KEY ( "no gold standard*"  OR  "without gold standard*"  OR  "missing gold standard*"  OR  "imperfect reference standard*"  OR  "no reference standard*"  OR  "missing reference standard*"  OR  "partial verification"  OR  "differential verification" )  AND  TITLE-ABS-KEY (“diagnostic accuracy”) AND TITLE-ABS-KEY  ( "medical test*"  OR  "new test*"  OR  "index test*"  OR  "diagnostic test*"  OR  "screening test*"  OR  routine* ) )  AND  ( LIMIT-TO ( PUBYEAR ,  2019 )  OR  LIMIT- TO ( PUBYEAR ,  2018 )  OR  LIMIT-TO ( PUBYEAR ,  2017 )  OR  LIMIT-TO ( PUBYEAR ,  2016 )  OR  LIMIT-TO ( PUBYEAR ,  2015 )  OR  LIMIT-TO ( PUBYEAR ,  2014 )  OR  LIMIT-TO ( PUBYEAR ,  2013 )  OR  LIMIT-TO ( PUBYEAR ,  2012 )  OR  LIMIT-TO ( PUBYEAR ,  2011 )  OR  LIMIT-TO ( PUBYEAR ,  2010 )  OR  LIMIT-TO ( PUBYEAR ,  2009 )  OR  LIMIT-TO ( PUBYEAR ,  2008 )  OR  LIMIT-TO ( PUBYEAR ,  2007 )  OR  LIMIT-TO ( PUBYEAR ,  2006 )  OR  LIMIT-TO ( PUBYEAR ,  2005)).

Similar search terms were used in other databases.
