# Supplementary material for: Diagnostic test evaluation methodology: A systematic review of methods employed to evaluate diagnostic tests in the absence of gold standard – An update
Source: PLoS One. 2019 Oct 11;14(10):e0223832. doi: 10.1371/journal.pone.0223832 (PMC6788703; doi:10.1371/journal.pone.0223832)
Supplement: S1 Supplementary Information — (DOCX) [file pone.0223832.s004.docx]

Supplementary Information

**Diagnostic test evaluation methodology: A systematic review of methods employed to evaluate diagnostic tests in the absence of gold standard – an update**

Table of Contents

[Introduction 3](#_Toc20992990)

[A1: Tables of methods employed in evaluating medical test(s) with missing gold standard in a binary-class diagnostic outcomes 4](#_Toc20992991)

[A2: Tables of methods employed to evaluate medical test when there is missing gold standard and the diagnostic outcomes is classified into three. Hence, focusing on ROC surface and volume of surface (VUS). 22](#_Toc20992992)

[A3: Tables of methods employed in evaluating medical test(s) with an imperfect reference standard or no gold standard. 25](#_Toc20992993)

[References 32](#_Toc20992994)

**List of tables**

[Table 1: Methods employed for single binary index test 4](#_Toc20993507)

[Table 2: Methods employed for multiple binary index tests 8](#_Toc20993508)

[Table 3: Methods employed for single ordinal index test 11](#_Toc20993509)

[Table 4: Methods employed for single continuous index test 14](#_Toc20993510)

[Table 5: Methods employed for single continuous index test with focus on covariate-specific ROC 19](#_Toc20993511)

[Table 6: Methods employed for multiple ordinal or continuous index tests 21](#_Toc20993512)

[Table 7: Methods employed for single ordinal index test with ROC surface and VUS 22](#_Toc20993513)

[Table 8: Methods employed for single continuous index test with ROC surface and VUS 22](#_Toc20993514)

[Table 9: Methods employed for multiple binary index tests and categorical disease status. 24](#_Toc20993515)

[Table 10: Methods employed to evaluate index test(s) when the diagnostic accuracy measures of the imperfect reference standard is known precisely. 25](#_Toc20993516)

[Table 11: Methods employed to evaluate index test(s) when the diagnostic accuracy measures of the imperfect reference standard is unknown. 26](#_Toc20993517)

[Table 12: Construction of reference standard. 30](#_Toc20993518)

[Table 13: Table of other methods employed to evaluate medical test(s) 31](#_Toc20993519)

## Introduction

In this supplementary material, the identified methods from the systematic review are briefly discussed. Some strengths and weaknesses of each method are highlighted. In addition, clinical applications (which are published articles where the method was employed) and key references (which are articles that described the proposed method) of the proposed methods are included.

As noted in the discussion section of the review, not all the methods have been applied outside the original publication of the developed method. Thus, those methods that have not been applied clinically outside the original publication of the developed method do not have clinical application.

The identified methods are presented in tables under three main indices (A1, A2 and A3):

- A1: include all methods proposed when the gold standard is missing for some of the participants in the study, and the diagnostic outcome is binary. This is from **Table 1** to **Table 6**. The tables are stratified based on the number of index test being evaluated and how the test outcome is measured (binary, ordinal or continuous).
- A2: includes all methods proposed to evaluate medical test with more than two diagnostic outcomes and the gold standard is missing for some of the participants in the study. **Table 7** to **Table 8** focuses on methods employed to estimate the on ROC surface and volume under the ROC surface of single index test with ordinal (**Table 7**) and continuous (**Table 8**) results; and **Table 9** focus on estimating the sensitivities and specificities of multiple binary tests when the disease status is categorical.

The methods listed under section A1 and A2 have the true disease status of some participants missing; hence, the methods are developed with the assumption that the missing disease status is either missing at random (MAR) or missing not at random (MNAR). And methods developed based on the assumption of missing not at random has the ‘missing at random’ as a special case.

- A3: includes methods proposed to evaluate medical test(s) when the reference standard is imperfect or there is no-gold standard. This is from **Table 10** to **Table 13**.

For all methods in A1, A2 and A3; when multiple tests are evaluated together, the tests could be conditional independent given the true disease status, or conditional dependent given the true disease status. Thus, some methods are developed based on these assumptions.

**Abbreviations**

CI: conditional independence LCM: latent class model

CD: conditional dependence WGEE: weighted generalised estimating equation

MAR: missing at random PG-BRL: partial gold Bayesian rank likelihood

MNAR: missing not at random DR: Doubly robust

AUROC: Area under the ROC curve VUS: volume under the surface

ROC: Receiver operating characteristic NI: non-ignorable verification

IPW: inverse probability weight EM: Expectation maximization

FI: Full imputation MSI: Mean square imputation

A1: Tables of methods employed in evaluating medical test(s) with missing gold standard in a binary-class diagnostic outcomes.

Table 1: Methods employed for single binary index test

| **Single binary index test** | | | | | | |
| --- | --- | --- | --- | --- | --- | --- |
| Method  (Year) | MAR / MNAR | CI / CD | Characteristics | Strength | Weaknesses | Key reference |
| Begg & Greenes (B&G)  (1983) | MAR | CI | This method is based on the Bayes theorem and the missing disease status of the unverified participants are considered as a doubling sampling problem. The true disease status of unverified participants are viewed as double-sampling problem and are included in the analysis.  **Clinical application:** [1-5] | - Easy to implement analytically and the results are easily interpretable. - Can adjust for binary or discrete covariates. | - Estimates of sensitivity and specificity could still be biased if there are few false negative in the study (Cronin and Vickers (6)). - In the presence of more than one covariate, parametric models could be considered. Hence, the estimates obtained could be prone to bias that arise from model-misspecification. | Begg and Greenes (7) |
| Likelihood-based  (1993) | MNAR | CI | The conditional probability of verification given the true disease status, are assumed to be known and specified with two values. Hence, the boundary of this values can be estimated using the observed data, which is employed to obtain bounded values for the sensitivity and specificity of the index test under the MNAR assumption. | - There is no need to conduct sensitivity analysis because this method produces all possible value of the sensitivity and specificity of the index test under the assumption that the verification is non-ignorable | - It assumes that the verification is known which is not always true in practice. - The estimated value of the sensitivity and specificity of the test is not a single value (except if the verification is specified) but a range of value that is bounded. Thus, interpretation of estimates is not straightforward. | Zhou (8) |
| Expectation maximization (EM) – based regression  (2003) | MNAR | CI | Parametric models are used to model the joint distribution of the verification, disease and index test response. The conditional probability of disease, test, and verification are modelled using logistic regression. The estimates of the marginal sensitivity and specificity are obtained by maximising the log-likelihood of the observed data using the EM approach. The MAR assumption is a special case of this approach. | - This method produces a single value each as the estimated sensitivity and specificity of the index tests not bounded values. - Observed discrete covariates can be considered with this approach. - Standard error of the parameters can be estimated using the observed information matrix not via bootstrapping. - The probability of verification is estimated from the observed data rather than assuming it is known. | - Due to the MNAR assumption, the model could be non-identifiable. Hence to ascertain if the estimates obtained are unique, the information matrix from the EM process need to be singular. - The choice of model-selection is limited because of the non-identifiability problem. - This method is prone to bias if the model is misspecified. | Kosinski and Barnhart (9) |
| Global sensitivity analysis  (2003) | MNAR | CI | This method provides all possible (but bounded) jointed values of the sensitivity and specificity of the index test under different assumptions of the missing mechanism (MCAR, MAR and MNAR) in a graphical form. The area produced by the bounded possible values is called the true ignorance region (TIR). It is a sensitivity analysis that help researchers see the amount of bias that can be made as a result of the MNAR assumption.  **Clinical application**: [10-13] | - This method allows researcher to understand how different missing mechanism assumption can impact their inference on the diagnostic accuracy of the index test. - The graphical presentation of all the possible pair values of the sensitivity and specificity makes the interpretation easy. - With this approach, there is no need to change the values of parameters in the fitted model to obtain all the possible pairs of sensitivity and specificity under the MNAR assumption. | - This approach does not take into consideration observed covariates such as age, race, etc. that could affect the verification of the true disease status of the participants. | Kosinski and Barnhart (14) |
| Multiple imputation  (2006) | MAR | CI | The true disease status of the unverified participants is considered as a missing data problem. So, the missing disease status are imputed with M (>1) plausible simulated values based on the assumption of MAR. M complete datasets are obtained. Each dataset is analysed to obtain the estimate of sensitivity and specificity of the test. The estimates are pooled together to obtain a single estimate of the sensitivity, specificity and its variances using the Rubin’s rule (Little and Rubin (15); Rubin (16)). | - This approach is an alternative to Beggs & Greenes method. However, it is shown to be more flexible than B&G in incorporating more than one covariates [17]. - Estimates obtained are easily interpretable. | - If the MAR assumption do not hold, the MNAR assumption need to be modelled within the data augmentation process which will require specifying the right model and could be computationally demanding. | Harel and Zhou (18) |
| Bayesian approach  (2006, 2008, 2018) | MAR & MNAR | CI | Generally, Bayesian method combines prior information about the parameters of interest such as sensitivity and specificity of the index test in a parametric distribution together with the likelihood of the observed data to get the predictive or posterior distribution which is used to make inference about the parameters of interest. The three articles discussed here estimates the test’s performance of a single test with binary response under MNAR and MAR assumption.  The method by Martinez (19) is the Bayesian approach of the likelihood – based method by Zhou (8). However, the verification quantities are taken as unknown and estimated alongside the sensitivity, specificity and prevalence of disease within the Bayesian process. This method does not employ observed covariates.  Buzoianu and Kadane (20) used the data augmentation approach (which is a Bayesian imputation approach) to impute the missing disease status and simultaneously estimate the parameters of the models which is used to obtain the marginal sensitivity and specificity of the index test. This is the Bayesian approach of Kosinski and Barnhart (9).  The Bayesian approach by Hajivandi, Shirazi (21) is an extension of the approach by Martinez (19) and Buzoianu and Kadane (20). This extended Bayesian method eliminates the verification variable. So that the probability of disease or test response do not depends on verification. Hence, a new model is developed to predict the true disease status for individuals who do not undergo the index test. | - Generally, Bayesian methods overcome the problem of model non-identifiability faced by the maximum likelihood approach. Because they use prior information about the parameters of interest which imposes some statistical restriction on the parameters. - The number of parameters to be estimated using Bayesian approach is not limited like the maximum likelihood approach. - Prior distribution of the sensitivity and specificity can be elicited from the estimates obtained from the B&G approach (Martinez (19)) or elicited from other sources like expert opinion or previous research. | - Sensitivity analysis is required to study the impact of the prior distribution on the estimates. - If the prior is non-informative then estimates tend towards likelihood. However, if informative priors are used, the obtained estimates tend to balance the idea. - Sufficient sample size is required to avoid the inference to coincide completely with the prior. | Martinez (19)  Buzoianu and Kadane (20)  Hajivandi, Shirazi (21) |
| Propensity score  (2012) | MAR | CI | This method defines the propensity score as “*the probability of verification given the test response and observed covariates*”. It is assumed to be unknown and estimates using the observed data. Participants are stratified based on their propensity score. Estimate of sensitivity and specificity of the index test are obtained by pooling the estimates obtained from each strata. | - Although, this approach is model-based, the estimates obtained are less sensitive to model misspecification because the propensity score (verification model) is used to stratify the participants. | - The number of strata has to be decided. - This method requires the sample size to be sufficiently large because of the stratification. | He and McDermott (22) |

Table 2: Methods employed for multiple binary index tests

| **Multiple binary index tests** | | | | | | |
| --- | --- | --- | --- | --- | --- | --- |
| Method | MAR / MNAR | CI / CD | Characteristics | Strength | Weaknesses | Key reference |
| Baker et al  (Maximum likelihood based)  (1995) | MNAR | CI | The idea behind this approach is to use multiple tests to fit identifiable verification models to overcome the problem of non-identifiability that arises as a result of the MNAR assumption in the maximization of the log – likelihood function. The diagnostic accuracy measures of the combined multiple tests is obtained by choosing the disease model and verification model that fits the data. This method derives the ROC curve using the combined responses of the multiple index tests employed in the study to obtain the combination of the tests’ response that maximizes TPRs at a given FPRs. | - The use of multiple index tests makes the likelihood of the observed data identifiable under the MNAR assumption. - The model can be extended to include covariates. | - This method does not estimate the diagnostic accuracy measures of the index tests individually but only collectively. - This method does not consider the conditional dependence of the multiple index tests. - The estimates obtained are sensitive to the MNAR assumption (via the verification model). Thus, they are prone to bias that could arise from model misspecification. Evidence of model misspecification can be checked by fitting various verification models, | Baker (23) |
| Maximum likelihood approach  (1998) | MAR | CI | This method uses the maximum likelihood approach to estimate the sensitivities and specificities of two binary (screening) index tests under the assumption that both tests are conditional independent given the true disease status. | - Estimates are easy to compute or solve analytically. - The method can incorporate binary or categorical covariate to obtain the covariate specific diagnostic accuracy measures. | - Method cannot be employed in the case of more than two index tests. - Tests must be conditional independent. | Zhou (24) |
| Latent class model  (LCM)  (1999, 2008) | MAR | CI & CD | When two screening tests are used the screen negatives (those participants with negative responses in both tests) are considered to be negative or non-diseased. Hence, they are not referred for further testing with the gold standard because their true disease status is assumed to be negative.  Methods (based on LCM) were developed by Walter (25), Böhning and Patilea (26) to estimate the sensitivities and specificities of the two binary index (screening) tests when all screen negatives do not get their disease status verified with the gold standard. The disease status of unverified participants are assumed to be latent or unobserved or missing but not negative. Because the two screening tests are imperfect.  Walter (25) assumes both tests have dichotomised responses and they are conditional independent given the true disease status.  Böhning and Patilea (26) is an extension of Walter (25) which relaxes the assumption of conditional independence and assumes that both tests are conditionally dependent given true disease status and that the conditional dependence is homogenous. | - The assumption that all participants with negative response in both tests are non-diseased is relaxed because both index (screening) tests are imperfect. - Sensitivities and specificities are calculated separately for the two tests evaluated. - Böhning and Patilea (26) do not require the two tests’ responses to be independent. | - The homogenous conditional dependence of the tests across all participants may not be true in practice. - The estimates obtained can be biased if the model is misspecified. | Walter (25)  Böhning and Patilea (26) |
| Bayesian approach  (2005) | MAR | CI | This Bayesian approach estimates the sensitivity and specificity of two index tests which are applied to all participants while adjusting for observed covariates. | - Using informative priors circumvent the problem of non-identifiability that arises from likelihood estimation procedure. - Correlation between tests is considered. | - Misspecification of the prior model or distribution can bias estimates. | Martinez, Achcar (27) |
| Lloyd et al  (2008) | MAR | NIL | This method uses multinomial logistic regression to estimate the joint sensitivity and specificity of all the index tests. The aim of this method is to evaluate the diagnostic accuracy of a combination of multiple dichotomised or binary tests to decide verification process. | - The conditional dependence or independence of the tests is of no importance in this approach. | - It does not provide the diagnostic accuracy of the individual index test applied in the study. | Lloyd (28) |
| Semi-latent class:  Gaussian random effect (GRE)  Finite mixture (FM)  (2008) | MAR | CI & CD | This approach is a modification of the GRE by [29] and Finite mixture by Albert and Dodd (30) which was original developed to evaluate medical tests when the is no gold standard or the reference test is imperfect. This method is semi-latent because the disease status of those that were verified with the gold standard are taken to be known and participants not verified are assumed to be latent. This method is an alternative to the imputation and reweighting approach by Albert (31). | - This is more robust than the imputation approach by Albert (31) if the models are correctly specified. - It is a data-driven method. | - Prone to bias that could arise from model misspecification. | Albert (32) |
| Imputation and reweighting:  MSI, IPW, and SPE  (2007) | MAR | CI & CD | This approach applies the idea of the estimators by Alonzo and Pepe (33) (MSI, IPW and SPE) to estimate the diagnostic accuracy measures of multiple binary tests. The joint and marginal sensitivities and specificities of the tests being evaluated are estimated under the assumption that the verification is known or fixed by design. However, if it isn’t, then it can be estimated using the observed data. This approach is an alternative to the semi-latent methods by Albert (32) | - Comparing this approach to the semi-latent methods (GRE and FM) by Albert (32), it is simple and less prone to errors due to model misspecification. - It is less computational expensive compared to the semi-latent methods. - Additional discussion about the advantages and disadvantages of the two methods are in this article. | - The assumption that the verification is being fixed by designed or known is very restrictive and may not always be true in practice. - In extreme biased sampling (those with all negative index test response do not get their disease status verified), the imputation approach requires statistical adjustment like extrapolation. | Albert (31) |
| Bayesian approach  (2010) | MNAR | CI | This is a modification of the Martinez (19) approach to include two index binary tests. It uses Markov – chain Monte Carlo (MCMC) methods to simulate samples for the joint posterior distribution. The probability of verification is modelled using eight parameters in the form described by Zhou (8). | - Employing two good sources of information rather than only the observed data can improve the accuracy of the estimates. | - Relatively large sample size is needed to reduce the weight of the prior on the posterior. | Aragon, Martinez (34) |
| Likelihood –based approach  (addresses double partial verification)  (2012) | MNAR | CI | This approach deals with double partial verification where not all the participants undergo all the index tests and some do not undergo the gold standard. It is likelihood-based following the pattern of Baker (23), and Kosinski and Barnhart (9). | - Rather than using only participants that undertook both index tests (probable discarding data from participants who did not undergo both index tests); this approach utilises every data from all participants whether they undertook either index tests. | - This method is model-based, so it is prone to bias due to model misspecification. - This method is computational expensive, especially when the number of index tests with conditional dependence increases and the number of missing participants within each tests varies. | Van Geloven (35)  Van Geloven, Broeze (36) |
| Weighted Generalise Estimating Equation (WGEE)  based method  (2014, 2003, 2006) | MAR | CD | WGEE is employed to deal with correlated data with missing data problem. Because not all participants undergo the gold standard the number of verified participants are used as the weight.  The single model proposed by Xue, Kim (37) estimates the diagnostic accuracy of multiple index tests and compare them. The model can also combines data from different study to obtain the sensitivity and specificity of the index tests.  The weighted least square (WLS)[38] method and WGEE method by Lin, Barnhart (39) are proposed to estimate the diagnostic accuracy of the index test when applied at subunit levels. | - This approach can estimates the diagnostic accuracy of more than two binary index tests that are correlated. - Data from two or more study can be combined using this approach to obtain the parameters of interest. | - This approach requires large sample size because of the normal approximation assumption. - It is model – based; hence it is prone to error due to misspecification of models. - With this approach, verification depends only on the test response not on the observed covariates. Thus, it cannot adjust for observed covariates. | Xue, Kim (37)  Barnhart and Kosinki (38)  Lin, Barnhart (39) |

Table 3: Methods employed for single ordinal index test

| **Single ordinal index test** | | | | | | |
| --- | --- | --- | --- | --- | --- | --- |
| Method | MAR / MNAR | CI / CD | Characteristics | Strength | Weaknesses | Key reference |
| Parametric AUROC  (1984) | MAR | CI | Constructing a ROC curve using the pair of sensitivities and specificities estimated using any correction method for binary test (like the B&G method) at every cut-off of continuous or ordinal tests often produce a step function ROC (not smooth). Hence, this method employs the procedure of Dorfman and Alf Jr (40) to derive a smooth ROC curve. The method assumes that the index test result has an underlying continuous scale; the disease and non-diseased group of the underlying continuous scale of the test response are normally distributed (bi-normality) after monotonic transformation, and there are latent cut-offs. With the ROC curve derived, the AUROC is calculated graphically or using the trapezoidal rule by Bamber (41). | - This method produces a smooth ROC and the AUROC is estimated graphically or via the trapezoidal rule. - Estimate is easy to interpret. | - The underlying bi-normality assumption of the disease and non-diseases group may not always be true in practice. - This approach estimates only the AUROC. - Pairs of sensitivity and specificity on the smooth ROC cannot be obtained at any cut-off because they are latent and are employed for model purpose. - With the derived ROC curve, the test’s response at the extreme of the upper ROC curve are indicative of non-diseased than diseased, which is not in practice. - The estimated AUROC depends on the number of verified participants; thus, a relative high sample size is advised. - This method can be prone to bias that arises from model-misspecification. - This method cannot incorporate any observed covariates as the verification of participants depends only on the test response. | Gray, Begg (42) |
| Non-parametric likelihood based  (1996) | MAR | CI | This method estimates the AUROC of an ordinal test via likelihood approach. | - This approach is non-parametric so not prone to model-misspecification. - No need to make any assumption about the distribution of the disease and non-disease group. - This approach can adjust for discrete covariates. | - This approach estimates only the AUROC, not the ROC curve (the pairs of sensitivities and specificities at different cut-offs). | Zhou (43) |
| Parametric maximum likelihood based  (1998) | MNAR | CI | This method employs the basic assumption of Dorfman and Alf Jr (40) procedure to derived a smooth ROC curve. The EM algorithm is employed to obtain the maximum likelihood estimator of the ROC curve. The method incorporates sensitivity analysis to evaluate the impact of the MNAR assumption. | - This approach produces range of possible values for the pair of sensitivity and specificity under the non-ignorable verification assumption. Hence, a separate sensitivity analysis is not needed. - If the verification is known and accurate, then the estimates obtained are robust. | - Assumption of bi-normality of disease and non-disease group is made which may not be true in practice. - The estimates of sensitivity and specificity is bounded by a range of values and not a single value. - The verification is assumed to be known, which may not be true in practice. | Zhou and Rodenberg (44) |
| Parametric maximum likelihood based  (2000) | MAR | CI | This approach is an extension of Gray, Begg (42) method to include observed categorical covariates that can affect the verification of the disease status of the participants. This approach calculate the AUROC and the ROC curve applying the procedure and basic assumption of Dorfman and Alf Jr (40). | - This method produces covariate-specific ROC and AUROC. | - The underlying assumption of bi-normality about the diseased and non-diseased group may not be true in practice. - This method is prone to bias that can arise from model – misspecification. - Pairs of sensitivity and specificity on the smooth ROC cannot be obtained at any cut-off because they are latent and employed for model development. | Rodenberg (45) |

Table 4: Methods employed for single continuous index test

| **Single continuous index test** | | | | | | |
| --- | --- | --- | --- | --- | --- | --- |
| Method | MAR / MNAR | CI / CD | Characteristics | Strength | Weaknesses | Key reference |
| Hunink et al  (I990) | MAR | CI | This approach evaluates the ROC curve of a continuous test based on the assumption that the selection for verification depend on observed covariates and not the result of the index test. Logistic regression analysis was employed to evaluate the probability of verification given the observed covariates to correct for verification bias. | - A single ROC curve that adjust for all the covariates employed in the study can be produced as well as covariate specific ROC curves. | - In practice, using only observed covariates such as signs and symptoms, age or sex may not be a strong evidence to send participants for disease verification. | Hunink, Richardson (46) |
| Imputation and Reweighting:  Full imputation (FI)  Mean score imputation (MSI)  Inverse probability weight (IPW)  Semi-parametric efficient (SPE)  (2005) | MAR | CI | The four estimators (FI, MSI, IPW and SPE) derive the ROC curve and AUROC from empirical data. The FI estimator imputes the probability of disease (disease status) for all participants in the study regardless if they were verified with the gold standard or not. The MSI imputes the probability of disease only for participants with missing disease status. The IPW uses participants whose true disease status were verified. It weights each observation from the verified with the inverse of its probability of verification. The SPE incorporate the probability of verification and probability of disease to obtain the estimates of sensitivity and specificity. That is why it is referred to as doubly robust. The AUROC is estimated using trapezoidal rule. A modification of the FI, MSI, IPW and SPE estimators that adjust for non-ignorable verification is constructed by Liu (47). | - No assumption is made about the distribution of the test response of the diseased and non-diseased group. - These methods are easy to implement, because the estimators only need regression model is fitted to the binary response – disease and or verification. - The four estimators are consistent, provided the disease model and/or verification is specified correctly. - The SPE is doubly robust; the correct specification of either the verification or disease model make it a consistent estimator. | - The estimators are model-based, so prone to bias due to model misspecification. The estimators are inconsistent if the model is misspecified. The effect of model misspecification on each of the estimator is discussed in the article. - Estimates of sensitivity and specificity could still be biased if there are few false negative in the study (Cronin and Vickers (6)). - The SPE estimator does not produce a monotonic (increasing) ROC. Although, this can be corrected using isotonic regression (Fluss (48)). - The SPE is inconsistent if both verification and disease model is misspecified. - If there are observed covariate(s) that affected verification, this approach does not adjust for it. - The IPW employs information from verified participants, hence there is loss of information from those unverified. | Alonzo and Pepe (33) |
| Doubly robust (DR):  AUROC only and  Empirical ROC  (2006, 2009) | MNAR | CI | The doubly robust estimators discussed here produces marginal AUROC and ROC not covariate specific ROCs and AUROCs. The disease status of all the participants (verified or unverified) are replaced with the estimated disease which is a function of the probability of disease and verification probability. Both probabilities are specified parametrically. It is doubly robust because the correct specification of either the disease or verification model makes the estimator consistent. However, misspecification of both models makes the estimator inconsistent. Sensitivity analyses are undertaken to evaluate the impact of the MNAR assumption on the estimates. | - The DR approaches are consistent and asymptotic normal provided the either the verification or diseased model is correctly specified. - This approach can adjust for continuous covariate without having to dichotomised or discretize them. - The number of covariates that can be included in the model is not limited. | - The estimator is model-based so misspecification of the models makes the estimator inconsistent. - There is efficiency cost with this approach; an estimator with correctly specified disease or verification model yields same consistent estimates as the DR estimator. However, the variance of the DR estimator is larger because of the additional model. - The non-ignorable parameter is assumed to be known which is not always true in practice. - The empirical ROC curve derived by Fluss (48) has non-monotonic property because the value of the ROC can lie outside the range of 0 and 1. Hence, the isotonic regression procedure is suggested to correct for the non-monotonicity of the ROC (estimated sensitivities and specificities). | Rotnitzky, Faraggi (49)  (AUROC only)  Fluss (48)  (Empirical ROC) |
| U-statistics estimator  (2009) | MAR | CI | The AUROC is estimated based on U-statistics and inverse probability weighting (IPW) technique. The IPW technique is used to correct for verification bias and the probability of verification is assumed to be known. The test response of the diseased and the non-diseased group are assumed to follow the F distribution and the U-statistics estimator is constructed under this assumption. | - The estimate obtained with this method is equivalent to the estimate obtained using the IPW estimator by Alonzo and Pepe (33). However, this estimator has a closed form variance. - This method does not require the probability of disease as it depends mainly on the verification probability. And if this is known, then it is less prone to bias that could arise from model misspecification. | - The assumption that the test’s response follows an F-distribution may not be true in practice. - This approach only estimates the AUROC not the ROC curve. - The verification probability may not be known in practice. | He (50) |
| Likelihood based (imputation and reweighted):  FI, MSI , IPW and Pseudo – DR (PDR)  (2010) | MNAR | CI | This approach is an extension of Alonzo and Pepe (33) to account for non-ignorable verification. The non-ignorable parameter was estimated from the observed data (rather than taken to be known / specified) by using the whole participants (not only those verified) to model the disease model. The log-likelihood is a function of both the disease and verification model. The log-likelihood was solved using scoring equations. Estimates of the probability of disease and verification probability is obtained which is employed to estimate the ROC curve and AUROC. The Pseudo DR is not doubly robust as the SPE estimator [33] or the DR estimators [48, 49]; because the verification probability and disease probability are estimated from same likelihood function and correct specification of both model is required to make the PDR estimator consistent. | - The estimators do not specify the NI parameter or assumes it to be known like the DR approach; rather it is estimated from the observed data. - The estimators derive both ROC curve and AUROC. - The disease’s estimator for the MSI and PDR methods are statistically unbiased. - The estimators are consistent provided the underlying assumptions surrounding their development are fulfilled. | - The misspecification of the models will cause the estimator to be inconsistent. - The PDR estimator produces non-monotonic ROC curve. However, this can be corrected using isotonic regression technique (Fluss (48)) - None of the estimators including the PDR estimator have the doubly robust property. - A reasonably large sample size is required because of the non-ignorable parameter that is estimated from the observed data. | Liu (47) |
| Partial gold Bayesian rank likelihood (PG-BRL)  (2014) | MAR | CI | This approach is a modification of Gu and Ghosal (51) originally developed to derive the ROC and AUROC of diagnostic tests with continuous response in full or complete verification of participants with the gold standard. The PG-BRL is constructed to correct for verification bias. The method uses Bayesian technique to estimate the posterior distribution of the bi-normal parameters (mean and variance) of the disease group only (because the non-disease group is assumed to follow the standard normal distribution) and the prevalence of the true disease status. The observed data are placed in rank and label. Labels are used to describe the true disease status of the verified participants (non-disease = 0 and disease – 1) and missingness of unverified participants (unverified = 2). The ranks are invariant. Due to the missing labels of some participants, the data argumentation technique is applied via Gibbs sampling to impute the missing labels. Inference about the ROC and AUROC are derived using the posterior distributions of the parameters estimated (mean and variance). | - This estimator derive both the ROC and AUROC under the bi-normality assumptions of the diseased and non-diseased group. - The PG-BRL estimator is consider to perform equivalently in terms of accuracy when compared to some bias – correction estimators like the FI, MSI, IPW and SPE [33]. | - The assumption of bi-normality of the disease and non-disease group after some transformation is a restrictive assumption in practice. - Adjusting for observed covariates with this approach is computational complex as various transformation of the data are required. - To use this approach for MNAR the verification needs to be explicit known and must be reflected in the verification function. | Gu, Ghosal (52) |
| K – nearest neighbour  (2015, 2017) | MAR | CI | These non-parametric estimators employs the K nearest neighbour imputation approach (Ning and Cheng (53)) to impute the missing disease status of participants where the diseases status is not verified. | - It is a fully non-parametric approach, so not prone to model misspecification. - It is a non-parametric version of the MSI approach of Alonzo and Pepe (33). - The estimator is consistent and asymptotically normal under MAR assumption. | - The choice of K and distance measure could be quite challenging in obtaining an unbiased estimate of the TPR and FPR. - This approach requires a reasonably high sample size. | Adimari and Chiogna (54)  (ROC)  Adimari and Chiogna (55)  (AUROC) |
| Propensity – score adjustment method  (2018) | MNAR | CI | It’s a parametric approach. The probability of verification for the diseased participants not the whole sample is model under some parametric assumption. The AUC of the index test is estimated. | - Since this method uses parametric assumption to model the probability of verification for only verified participants than the whole sample; the approach seems to be quite simpler or straightforward. | - The parametric assumption used to model the probability of verification for the verified participants could be misspecified or non-ideal in practice; thus, resulting in wrong estimated of the AUC. | Yu, Kim (56) |

Table 5: Methods employed for single continuous index test with focus on covariate-specific ROC

| **Single index test with continuous response but focus on covariate specific ROC** | | | | | | |
| --- | --- | --- | --- | --- | --- | --- |
| Method | MAR / MNAR | CI / CD | Characteristics | Strength | Weaknesses | Key reference |
| Fully parametric  (2009) | MNAR | CI | This method is a variation of the DR approach by Rotnitzky, Faraggi (49). However, the joint distribution of the test, disease, observed covariates and verification are specified parametrically rather than using the likelihood of the joint distribution. The ROC curve and AUROC derived are covariate specific. The observed covariate can be in continuous form. | - The estimator is consistent provided the underlying assumptions involving their development is fulfilled. - The number of covariates are unlimited and the continuous form of the covariates are retained. | - Requires large sample size. - Model – misspecification makes the estimator inconsistent. | Page and Rotnitzky (57) |
| Semi- parametric:  FI, IPW and PDR  (2011) | MAR | CI | The three estimators proposed here are modifications of the imputation and reweighting approach of Alonzo and Pepe (33) and doubly robust approach by Rotnitzky, Faraggi (49) to produce ROC curves that are covariate specific. | - Compared to the fully parametric approach [57], the semi-parametric estimators is less impacted by model-misspecification. | - This approach only produces ROC curves but not AUROC because it does not have an explicit expression. - The continuous covariates has to be change to discrete form. | Liu (58) |
| Doubly Robust  (2012) | MNAR | CI | This method is an extension of the DR estimator by Fluss (48) to make the estimated pair of sensitivity and specificities (ROC) covariate specific. To achieve this, semi-parametric location scale model was used to model the effect of the observed covariates on the ROC curve. The location scale model models the test’s response as function of the disease and covariates. | - The estimator is doubly robust - It is consistent and asymptotically normal. - The covariate is in discrete form. | - Estimator is inconsistent if the both models (verification and disease) are misspecified. - The derive ROC is non – monotonic so the isotonic regression corrected is needed - The non-ignorable parameter is specified so sensitivity analysis is required to study the impact of the MNAR assumption. | Fluss, Reiser (59) |
| Imputation & reweighting  (2013) | MAR & MNAR | CI | This method is a modification of the semi-parametric method by Liu (58) to develop an estimators that can also estimates the covariate – specific AUROCs | - The method also estimates the AUROCs unlike the semiparametric approach. - Adjust for covariates without having to change to discrete form. | - It is model-based, hence the estimator is inconsistent in the presence of model misspecification. | Liu (60) |

Table 6: Methods employed for multiple ordinal or continuous index tests

| **Multiple index tests with ordinal or continuous responses** | | | | | | |
| --- | --- | --- | --- | --- | --- | --- |
| Method | MAR / MNAR | CI / CD | Characteristics | Strength | Weaknesses | Key reference |
| Profile and EM method  (2003) | MNAR | CI | This is a ML – based approach which employs the profile method combined with EM to obtain a global maximum likelihood estimator for the sensitivity and specificity of two index tests with ordinal results. Although the article goes further to compare the two tests with assumption that they are correlated. However, this review focused on the approach employed to estimate the AUC of each index test. | - This approach is non-parametric, so not prone to model misspecification. | - In estimating the AUC of each index test, the two tests are assumed to be independent given the true disease status. - The two index tests must have the same number of ordinal class. | Zhou and Castelluccio (61) |
| Likelihood based Imputation  (MCEM) approach  (2012) | MAR | CD | This estimator is developed to estimate the ROC curve and AUROC of diagnostic tests in a multi-phase trial with partial verification. That is a trial with more than two – phase. The gold standard is applied to participants in the last phase of the trial to confirm true disease status. In multi-phase trial with multiple screening tests, decision rule is made on how to define positive and negative to move participants unto the last testing stage. Often times, the tests could be correlated or repeated. This method derived two estimators of the ROC curve when the believe-the-positive (BP) decision rule is used or believe-the-negative (BN) rule is employed. It is a likelihood-based approach and the Monte Carlo Expectation Maximization (MCEM) is used to maximise the log-likelihood. | - This method estimates the diagnostic accuracy of screening (index) tests that could be correlated and are employed in multi-phase trials which adjusting for partial verification within each phase. | - This method is model-based hence model misspecification makes the estimator inconsistent. - The method is computational complex as the MCEM is employed because direct maximization approach is difficult. - As the number of sequential tests employed in the study increases, the more complex the computational procedure of estimating the diagnostic accuracy especially if the tests are conditional dependent given true diseases status. | Yu (62) |

## A2: Tables of methods employed to evaluate medical test when there is missing gold standard and the diagnostic outcomes is classified into three. Hence, focusing on ROC surface and volume of surface (VUS).

Table 7: Methods employed for single ordinal index test with ROC surface and VUS

| Single ordinal index test | | | | | | |
| --- | --- | --- | --- | --- | --- | --- |
| Method | MAR / MNAR | CI / CD | Characteristics | Strength | Weaknesses | Key reference |
| Non-parametric likelihood-based approach  (2008) | MAR | CI | This estimator estimates the empirical ROC surface and volume under the ROC surface (VUS) using likelihood – based approach. | - This estimator is not model-based so bias due to model misspecification is eliminated. - Covariates can be adjusted; however, it must be in the discrete form to get covariate specific ROC surface. | - Sparse data affects the estimate derived. | Chi (63) |

Table 8: Methods employed for single continuous index test with ROC surface and VUS

| Single continuous index test | | | | | | |
| --- | --- | --- | --- | --- | --- | --- |
| Method | MAR / MNAR | CI / CD | Characteristics | Strength | Weaknesses | Key reference |
| Imputation and reweighting: MSI, FI, IPW and SPE  (2016) | MAR | CI | This estimator is an extension of the imputation and reweighting approach (FI, MSI, IPW and SPE) by Alonzo and Pepe (33) to incorporate three disease class status. | - Same as the imputation and reweighting estimators of Alonzo and Pepe (33). | - Same as the imputation and reweighting estimators of Alonzo and Pepe (33). | Duc, Chiogna (64) |
| Non-parametric approach: KNN approach  (2016, 2017) | MAR |  | This estimator is an extension of the KNN approach by Adimari and Chiogna (54). | Same as the KNN approach above. | Same as the KNN approach above. | Duc, Chiogna (65)  To Duc (66) |
| IPW  (2016) | MAR | CI | This estimator is an extension of the IPW approach by Alonzo and Pepe (33) which both derive the ROC surface and estimate the VUS. Only information from verified participants is employed in the analysis and each observation from verified participant is weighted with the inverse of the verification probability. | - The estimator is consistent given the verification of probability is accurately known or estimated. | - There is loss of information especially from participants whose disease status were not verified with the gold standard. - Same as the IPW estimator above. | Zhang, Alonzo (67) |
| IPW, DR and PDR estimators  (2018) | MNAR | CI | Three estimators are developed to estimate the volume under the ROC surface (VUS) only. These estimators are extension of the IPW estimator under the framework of the doubly robust technique, the DR and PDR approach by Rotnitzky, Faraggi (49) and Liu (47) respectively. | - The IPW and DR correct for verification bias in considerable samples; however the PDR approach requires large sample. - The DR estimator has the doubly robust property. - The PDR has same strength and weaknesses as above. | - Sensitivity analysis is required to study the effect of specifying the non-ignorable parameter in the DR and IPW approach. | Zhang, Alonzo (68) |
| Bayesian Semi-parametric ROC  (2018) | MAR | CI | The method is based on tri-normality assumption and it is an extension of the rank-based likelihood approach by Gu and Ghosal (51), Gu, Ghosal (52) | - This method uses the tri--normality assumption to model the three diagnostic outcomes, resulting in a smoother ROC surface. | - Adjusting for observed covariates is computational complex. - It is a parametric approach, so deviation from the parametric assumptions like the tri-normality of the diagnostic outcomes leads to inconsistent estimators. | Zhu and Ghosal (69) |
| Parametric –based approach  FI, MSI, PDR  (2019) | MAR / MNAR | CI | Parametric regression model is used to model the probability of disease and verification using the whole sample and not only participants whose disease status were verified with the gold standard. It is an extension of the approach by Liu (47). | - The NI parameter is estimated from the observed data. - The estimators are consistent provided the underlying assumptions surrounding their development are fulfilled. | - The misspecification of the models will cause the estimator to be inconsistent. - The PDR estimator has the doubly robust properties if either the disease or verification model is specified correct under the MAR assumption. However, under the MNAR assumption the PDR estimator needs both the model to be correctly specified to be consistent. | To Duc, Chiogna (70) |

Table 9: Methods employed for multiple binary index tests and categorical disease status.

| Multiple binary tests and categorical disease status | | | | | | |
| --- | --- | --- | --- | --- | --- | --- |
| Method | MAR / MNAR | CI / CD | Characteristics | Strength | Weaknesses | Key reference |
| Latent class model  (LCM)  (2010) | MAR | CI & CD | Chu (71) proposed a LCM method (frequentist and Bayesian) to estimate the sensitivities and specificities of the two index (screening) tests when all participants with negative responses in both tests do not get their disease status verified with the gold standard. The disease status of unverified participants is assumed to be latent or unobserved. The tests are binary, but the disease status is categorical. This approach assumes that only participants with negative results in the two binary tests applied do not get their disease status verified. | - The Bayesian approach overcomes the non-identifiability problem encountered using the frequentist approach. | - The conditional dependence model is not robust in that different conditional dependent homogenous model produces different estimates of the diagnostic accuracy measures and all fir the data. | Chu (71) |

##

## A3: Tables of methods employed in evaluating medical test(s) with an imperfect reference standard or no gold standard.

Table 10: Methods employed to evaluate index test(s) when the diagnostic accuracy measures of the imperfect reference standard is known precisely.

| **Methods** | **Characteristics** | **Strengths** | **Weakness** | **Key reference** |
| --- | --- | --- | --- | --- |
| Algebraic correction functions  (1966, 1981, 1996)  Bounded algebraic correction function  (1981, 2018) | The estimators considered here are mathematical functions. The bias-corrected sensitivity and specificity of the new test is a function of the known sensitivity and specificity of the imperfect reference test. These methods are applied to evaluate medical test with binary response.  The approach by Emerson, Waikar (72) aim to estimate the possible minimum and maximum values of the sensitivity and specificity of the index test when evaluated using an imperfect reference standard and the diagnostic accuracy of the reference standard is known.  **Clinical Application**:  Hahn (73), Matos (74), Mathews, Cachay (75) | - It is easy to implement analytically. - Incorporating information from a reliable source in addition to the available data can improve the accuracy of the estimated parameters. - With the bounded correction by Emerson, Waikar (72), there is no need to perform sensitivity analysis as this approach provides the lower and upper bound value of the sensitivity and specificity of the test being evaluated. | - If the information about the diagnostic accuracy of the imperfect test is not accurate then more bias is produced. - The approach by Emerson, Waikar (72) estimates the upper and lower bounds of the sensitivity and specificity of the test being evaluate but not an exact single value. | Gart and Buck (76)  Brenner (77)  Staquet, Rozencweig (78)  Emerson, Waikar (72) |
| Gaussian Random Effect (GRE)  Finite Mixture (FM), and Beta Binomial  (2009) | These methods are model based. They are applied when there are multiple binary index tests to evaluate. These methods estimate the joint sensitivity and specificity of the index tests as well as their individual sensitivity and specificity while taking into consideration the conditional dependence structure across the index tests. The methods are modification of the GRE approach by Qu, Tan (29) and FM by Albert and Dodd (30). | - Can be applied to multiple binary tests with conditional dependence structure and conditional independence of the tests is a special case of this approach. - This approach is an alternative to the LCA method. - The estimates obtained from this approach can be very robust provided the diagnostic accuracy of the RS is high and the dependence structure among the index tests given the RS is also high. | - Misspecification of the model can bias the estimate obtained. - Inaccurate error rate of the imperfect tests can bias the estimates obtained. Thus, it is encouraged to obtain the accuracy measures of the imperfect reference standard in comparison with the GS (or participants with known disease status). | Albert (79) |

Table 11: Methods employed to evaluate index test(s) when the diagnostic accuracy measures of the imperfect reference standard is unknown.

| **Methods** | **Characteristics** | **Strengths** | **Weaknesses** | **Key reference** |
| --- | --- | --- | --- | --- |
| Discrepancy analysis | All participants undergo both the index test and a reference standard which is imperfect. Then participants with discordant responses undergo another test called the resolver test, which is not a gold standard but assumed to have a high accuracy.  This approach has been modified recently in that some samples with concordant responses are also verified with the resolver test or all positive responses to the index test and discordant responses are retested with the resolver test [80]. Another modification is by Hawkins (81) where participants that undergo the resolver test are sampled from the four groups (TP, FP, TN, FN) and the proportion of the verified and unverified samples are taken into consideration when estimating the sensitivity and specificity.    **Clinical application**  Van Dyck, Buvé (82); Juhl (80); Rostami (83)  Spada (84); Brocchi, Bergmann (85) | - Easy to implement analytically. - It is less expensive compared to where all participants have to undergo the three tests. | - This method has been shown to be biased; overestimating the sensitivity of the index test. | Hadgu (86)  Hadgu, Dendukuri (87)  Schiller (88)  Hawkins (81) |
| Latent class model (LCM) | This approach assumes the disease status of all the participants are latent (unobserved) no test is a reference standard as. All tests are evaluated using statistical model. It is often assumed that the tests’ responses are dichotomous. However, methods have been developed to derive the ROC and AUROC of medical tests when there is no gold standard.  LCM can be broadly divided into frequentist and Bayesian approaches. | - LCM can be applied to study whose diagnostic outcome (disease status) is dichotomous or more. Unlike the discrepancy approach, correction methods which is applied to studies with only binary diagnostic outcomes. - It evaluates all the tests employed in the study simultaneously, because none of the test employed in the study is a gold standard. | - Under the assumption that the tests are conditional dependent given the true disease status; the conditional dependence structure among the tests lacks robustness in that different dependence structure models fits the data but yield different estimates. - LCM are probability models that rely on parametric assumptions. |  |
| Frequentist LCM  (Binary or dichotomized tests) | Frequentist LCMs use information from the observed data only to compute the estimate of the diagnostic accuracy measures such as the sensitivity, and specificity of the index test.  **Examples of frequentist LCMs**   - ***Standard / Traditional two class latent class model (TLCM)*** by Hui and Zhou (89),[90] assumes the tests are conditional independent (CI) and their sensitivities and specificities are constant across all population.   **Clinical application of the TLCM**  [73, 91-97]   - ***MECM approach***: Kang, Carter (98) applied binary, independent assumption - ***Log-linear latent class model (LLCM)*** (Hagenaars (99)) relaxes the CI assumption of the traditional LCM and models conditional dependence using log-linear models. - ***Probit latent class model (PLCM)*** (Uebersax (100)) relaxes the CI assumption and assumes that the K latent classes follow some multi-variate normal distribution with a mean vector and covariance matrix. - Xu (101) ***extended LLCM and PLCM*** to take into consideration intermediate responses of the tests if they are available. Hence, the diagnostic outcome is not dichotomized but three-classed. - ***Gaussian random effect*** (GRE) ***LCM*** by Qu, Tan (29) estimates the diagnostic accuracies of multiple tests (or raters) and uses the Gaussian distribution to model the conditional dependence structure across the tests.   **Clinical application of the RE LCM**: [102-105]   - ***Finite mixture (FM) model*** by Albert, McShane (106) estimates the accuracies of multiple tests but use finite mixture to model the conditional dependence across the tests.   GRE and FM are subject specific, and the test sensitivity and specificity are fixed.   - ***Two-cross random effect LCM*** by Zhang (107) estimates the accuracies of multiple tests with conditional dependence by using the Monte Carlo Expectation Maximization (**MCEM**) algorithm to maximize the full likelihood of the data.   **Clinical application of two-cross random effect LCM**  Xie (108)   - Alternative to the MCEM two-cross RE is the ***maximum pseudo-likelihood estimation via Newton Raphson (NR) algorithm*** by Liu (109). This approach aims to reduce the computational time of two-cross RE especially if there are many participants and tests by constructing some class of pseudo-like function (pairwise likelihood, triple wise likelihood, hybrid likelihood, and dimensional-wise likelihood) which can be maximized using the expectation maximization (EM) or Newton Raphson algorithm. - The latent class model developed by Xue, Oktay (110) estimates the accuracy of two tests (with conditional dependence) specifically designed to diagnosis tumor mutations (molecular testing). It is a modification of the Random effect LCM by Qu, Tan (29) | - This approach is based on the observed data only. - Some of the frequentist approach are straight forward like the TLCM, LLCM; however, some are computational complex because of the conditional dependence structure across the tests like the PLCM, two-random effect LCM, - Some Latent class models have some advantages over the others as well as some disadvantages; thus, it is worth reading through the original articles where the models were proposed to select which models is appropriate for the analysis. | - Choice of model are restricted because of non-identifiability problem. Hence, strict assumptions are made to make the models identifiable. - The estimates obtained from the LLCM approach is not interpretable as the diagnostic accuracy of the tests evaluated. | Hui and Zhou (89); |
| Bayesian LCM  (binary or dichotomized tests) | Bayesian LCM combines the likelihood of the observed data with prior information about the parameters to be estimated (sensitivity and specificity). The Bayesian approach was developed to make the LCMs identifiable while accurately estimating the diagnostic accuracy measures.  **Examples of Bayesian methods**  Joseph (111), Dendukuri (112), Johnson, Gastwirth (113); Georgiadis, Johnson (114); Nérette, Stryhn (115); Dendukuri, Hadgu (116); Dendukuri (117) (multi-latent variable model); The MLVM uses both CI and CD assumptions. Martinez (118); Zhang (119); Dufour (120); García Barrado, Coart (121); Lu, Dendukuri (122)  **Clinical Application of different Bayesian methods:** [123-141] | - Bayesian LCM overcomes the problem of non-identifiability faced by the frequentist approach because they use probability constraints (called prior distribution) on the parameters to be estimated. Using an informative prior is recommended to make the model identifiable. - It is rational to assume that combining information from two reliable source tend to produce accurate estimate and inference. | - LCMs with different conditional dependence structures can produce different estimates and still fit same data. - To reduce the impact of the prior information on the estimates being derive, it is rational to have sufficient data size. - It is recommended that accurate and precise priors be used to yield accurate posterior inference. However, imprecise priors can be used provided they are initialize with accuracy (McDonald and Hodgson (142)). | Branscum, Gardner (143)  Berkvens, Speybroeck (144) |
| ROC curve approaches | Estimating the ROC curve using the standard latent class approach [89] for every cutoff and then plotting the curve using estimates obtained from this approach produces a ROC curve that is not monotonic [145].  However, there are methods developed to produce ROC curves in the case of imperfect reference test. They are:   - **ROC with ordinal tests**   Henkelman, Kay (146); Beiden, Campbell (147); Zhou (148); Wang, Zhou (149) is an extension of [148] to incorporate multiple tests; and Wang (150) is an extension of Wang, Zhou (149) to incorporate conditional dependence structure across the multiple tests being evaluated.   - **ROC with continuous tests**   Choi, Johnson (151); Wang (152); Branscum (153); Jafarzadeh (154); Hall and Zhou (155); Erkanli (156) | - This approach estimates the overall diagnostic accuracy of the test(s) being evaluated and does not assume that the test(s) being evaluated is dichotomized. | - Some of the methods are model-based so adequate specification of the model is important to derive unbiased estimates. - The methods are computational tasking especially when there are multiple tests to evaluate and the assumption of conditional dependence is imposed on the tests being evaluated. |  |

Table 12: Construction of reference standard.

| **Methods** | **Characteristics** | **Strength** | **Weakness** | **Key reference** |
| --- | --- | --- | --- | --- |
| Composite reference standard  (CRS) | CRS uses a predetermined rule to construct a reference standard test using multiple imperfect tests. The participants undergo all the tests (index test and the imperfect reference tests). The reference standard is formed using the responses from the imperfect tests excluding the index test. The new test is evaluated in comparison to the established reference test. The dual CRS (dCRS) proposed by Tang, Hemyari (157), employs the “any positive” rule and “all positive” rule to estimate the sensitivity and specificity of the index test.  **Clinical application:** [158-161] | - Combining different imperfect tests using a predetermined rule to rule in or rule out diagnosis. | - Can have incorporation bias if the index test is also part of the test employed to construct the reference standard. Paine, Basu (162) - Challenges can arise in deciding the number of tests to combine to make the constructed reference standard adequate to discriminate patients or participants with the target condition. - It could be burdensome, especially if the number of tests to combine is many. - The performance of this approach depends on the performance of each test employed as reference standard and the conditional dependence between the tests. | Schiller (88)  Naaktgeboren, Bertens (163)  Tang, Hemyari (157) |
| Expert or panel or consensus opinion | This approach employs the decision(s) of expert(s) of a health condition (disease) as the reference standard to evaluate the new or index test. Often, observed covariates like signs and symptoms of the participants or test response of another test (not the index test) can be used together with expert(s) decisions to ascertain the disease status of the participants.  **Clinical application**:[160] | - The accuracy of the experts can be close to gold standard because they have good knowledge of the target condition. | - There could be discrepancy across the experts’ decisions in confirm the diagnosis of the participants. - This approach could be time-consuming especially if there are large number of participants. | Bertens, Broekhuizen (164) |

Table 13: Table of other methods employed to evaluate medical test(s)

| **Method** | **Characteristics** | **Strengths** | **Weaknesses** | **Key reference** |
| --- | --- | --- | --- | --- |
| Study of agreement | The study of agreement looks at how two or more test responses agree or disagree. Often, this approach is used alongside other types of methods like the latent class analysis. Commonly used agreement measures in a diagnostic accuracy study is the Kappa statistic [165] (Cohen kappa and Fleiss or Scott Kappa) and McNemar test [166].  **Clinical application**:[124, 167, 168] | - It is a way to explore the observed data to understand the relationship between the tests’ responses. | - Using the approach as a measure of accuracy is not encouraged; because the disagreement or agreement between two or more tests does not imply that one test is better or more efficient than the other. | Zaki, Bulgiba (169) |
| Validation | With this approach the disease status of the participants in the study are known (case and control). The estimates obtained are often referred to as analytical sensitivity and specificity [170]. This approach assesses the test based on what it is supposed or designed to do.  **Clinical application**: [85, 171-174] | - This approach provides a basic knowledge on the performance of the index test. The estimates can be employed as prior information in Bayesian analysis when the test is applied to participants with unknown disease status. | - In practice, the diagnostic test is designed to be applied to participants who may have or may have not shown signs or symptoms of the disease; thus, the estimated diagnostic accuracy using this approach may not reflect the true diagnostic accuracy of the index test. | Elliott, Applegate (170) |
| Test positivity rate | This approach estimates the proportion of participants who have positive result in a test. It is often taken that a test with the highest positivity rate compared to other tests have better accuracy[159]. However, this may not be true because these tests are prone to misclassification error (that is they are not gold standards) and the number of participants with positive result could depend on the prevalence of the target condition in the sample (sub-population) that is being studied.    **Clinical application**: [82, 159, 167] | - This approach gives possible estimates of the sensitivities of the tests employed in the study. - It has been used to assess whether to include or exclude some tests in diagnostic accuracy studies (Van Dyck, Buvé (82)). | - This approach should not be used as a standalone analysis to decide the accuracy of index test; because having a positive result to an index test does not imply presence of target condition especially if the test is imperfect. |  |

# References

1. Bielak LF, Rumberger JA, Sheedy Ii PF, Schwartz RS, Peyser PA. Probabilistic model for prediction of angiographically defined obstructive coronary artery disease using electron beam computed tomography calcium score strata. Circulation. 2000;102(4):380-5. doi: 10.1161/01.CIR.102.4.380.

2. Capelli GN, A.; Nardelli, S.; di Regalbono, A. F.; Pietrobelli, M. Validation of a commercially available cELISA test for canine neosporosis against an indirect fluorescent antibody test (IFAT). Preventive Veterinary Medicine. 2006;73(4):315-20. doi: 10.1016/j.prevetmed.2005.10.001. PubMed PMID: WOS:000236336000007.

3. Ferreccio C, Barriga MI, Lagos M, Ibáñez C, Poggi H, González F, et al. Screening trial of human papillomavirus for early detection of cervical cancer in Santiago, Chile. International Journal of Cancer. 2012;132(4):916-23. doi: 10.1002/ijc.27662.

4. Iglesias-Garriz I, Rodríguez MA, García-Porrero E, Ereño F, Garrote C, Suarez G. Emergency Nontraumatic Chest Pain: Use of Stress Echocardiography to Detect Significant Coronary Artery Stenosis. Journal of the American Society of Echocardiography. 2005;18(11):1181-6. doi: <https://doi.org/10.1016/j.echo.2005.07.020>.

5. Punglia RS, D'Amico AV, Catalona WJ, Roehl KA, Kuntz KM. Effect of verification bias on screening for prostate cancer by measurement of prostate-specific antigen. New England Journal of Medicine. 2003;349(4):335-42.

6. Cronin AM, Vickers AJ. Statistical methods to correct for verification bias in diagnostic studies are inadequate when there are few false negatives: A simulation study. BMC Medical Research Methodology. 2008;8. doi: 10.1186/1471-2288-8-75.

7. Begg CB, Greenes RA. Assessment of diagnostic tests when disease verification is subject to selection bias. Biometrics. 1983;39(1):207-15.

8. Zhou XH. Maximum likelihood estimators of sensitivity and specificity corrected for verification bias. Communications in Statistics - Theory and Methods. 1993;22(11):3177-98. doi: 10.1080/03610929308831209.

9. Kosinski AS, Barnhart HX. Accounting for nonignorable verification bias in assessment of diagnostic tests. Biometrics. 2003;59(1):163-71. doi: 10.1111/1541-0420.00019.

10. van Geloven N, Broeze KA, Opmeer BC, Mol BW, Zwinderman AH. How to deal with double partial verification when evaluating two index tests in relation to a reference test? Statistics in medicine. 2012;31(11-12):1265-76.

11. Nishikawa H, Imanaka Y, Sekimoto M, Hayashida K, Ikai H. Influence of verification bias on the assessment of MRI in the diagnosis of meniscal tear. American Journal of Roentgenology. 2009;193(6):1596-602.

12. Nishikawa H, Imanaka Y, Sekimoto M, Ikai H. Verification bias in assessment of the utility of MRI in the diagnosis of cruciate ligament tears. American Journal of Roentgenology. 2010;195(5):W357-W64.

13. Ahmadi F, Rashidy Z, Haghighi H, Akhoond M, Niknejadi M, Hemat M, et al. Uterine cavity assessment in infertile women: Sensitivity and specificity of three-dimensional Hysterosonography versus Hysteroscopy. Iranian journal of reproductive medicine. 2013;11(12):977.

14. Kosinski AS, Barnhart HX. A global sensitivity analysis of performance of a medical diagnostic test when verification bias is present. Statistics in Medicine. 2003;22(17):2711-21. doi: 10.1002/sim.1517.

15. Little RJA, Rubin DB. The analysis of social science data with missing values. Sociological Methods & Research. 1989;18(2-3):292-326.

16. Rubin DB. Multiple imputation after 18+ years. Journal of the American statistical Association. 1996;91(434):473-89.

17. de Groot JAH, Janssen KJM, Zwinderman AH, Bossuyt PMM, Reitsma JB, Moons KGM. Correcting for Partial Verification Bias: A Comparison of Methods. Annals of Epidemiology. 2011;21(2):139-48. doi: 10.1016/j.annepidem.2010.10.004. PubMed PMID: WOS:000286348200009.

18. Harel O, Zhou XH. Multiple imputation for correcting verification bias. Statistics in medicine. 2006;25(22):3769-86.

19. Martinez EZAA, J.; Louzada-Neto, F. Estimators of sensitivity and specificity in the presence of verification bias: A Bayesian approach. Computational Statistics and Data Analysis. 2006;51(2):601-11. doi: 10.1016/j.csda.2005.12.021.

20. Buzoianu M, Kadane JB. Adjusting for verification bias in diagnostic test evaluation: A Bayesian approach. Statistics in Medicine. 2008;27(13):2453-73. doi: 10.1002/sim.3099.

21. Hajivandi A, Shirazi HRG, Saadat SH, Chehrazi M. A Bayesian analysis with informative prior on disease prevalence for predicting missing values due to verification bias. Open Access Macedonian Journal of Medical Sciences. 2018;6(7):1225-30. doi: 10.3889/oamjms.2018.296.

22. He H, McDermott MP. A robust method using propensity score stratification for correcting verification bias for binary tests. Biostatistics. 2012;13(1):32-47. doi: 10.1093/biostatistics/kxr020.

23. Baker SG. Evaluating multiple diagnostic tests with partial verification. Biometrics. 1995;51(1):330-7. doi: 10.2307/2533339.

24. Zhou XH. Comparing accuracies of two screening tests in a two-phase study for dementia. Journal of the Royal Statistical Society Series C: Applied Statistics. 1998;47(1):135-47.

25. Walter SD. Estimation of test sensitivity and specificity when disease confirmation is limited to positive results. Epidemiology. 1999:67-72.

26. Böhning D, Patilea V. A capture–recapture approach for screening using two diagnostic tests with availability of disease status for the test positives only. Journal of the American Statistical Association. 2008;103(481):212-21.

27. Martinez EZ, Achcar JA, Louzada-Neto F. Bayesian estimation of diagnostic tests accuracy for semi-latent data with covariates. Journal of Biopharmaceutical Statistics. 2005;15(5):809-21.

28. Lloyd CJF, Donald J. An application of multinomial logistic regression to estimating performance of a multiple-screening test with incomplete verification. Journal of the Royal Statistical Society: Series C (Applied Statistics). 2008;57(1):89-102. doi: 10.1111/j.1467-9876.2007.00602.x.

29. Qu Y, Tan M, Kutner MH. Random effects models in latent class analysis for evaluating accuracy of diagnostic tests. Biometrics. 1996;52(3):797-810. doi: 10.2307/2533043. PubMed PMID: WOS:A1996VF83800002.

30. Albert PS, Dodd LE. A Cautionary Note on the Robustness of Latent Class Models for Estimating Diagnostic Error without a Gold Standard. Biometrics. 2004;60(2):427-35. doi: 10.1111/j.0006-341X.2004.00187.x.

31. Albert PS. Imputation Approaches for Estimating Diagnostic Accuracy for Multiple Tests from Partially Verified Designs. Biometrics. 2007;63(3):947-57. doi: 10.1111/j.1541-0420.2006.00734.x.

32. Albert PSD, L. E. On estimating diagnostic accuracy from studies with multiple raters and partial gold standard evaluation. Journal of the American Statistical Association. 2008;103(481):61-73. doi: 10.1198/016214507000000329. PubMed PMID: WOS:000254311500014.

33. Alonzo TA, Pepe MS. Assessing accuracy of a continuous screening test in the presence of verification bias. Journal of the Royal Statistical Society: Series C (Applied Statistics). 2005;54(1):173-90. doi: 10.1111/j.1467-9876.2005.00477.x.

34. Aragon DC, Martinez EZ, Alberto Achcar J. Bayesian estimation for performance measures of two diagnostic tests in the presence of verification bias. Journal of Biopharmaceutical Statistics. 2010;20(4):821-34. doi: 10.1080/10543401003618868.

35. Van Geloven NB, K. A.; Opmeer, B. C.; Mol, B. W.; Zwinderman, A. H. How to deal with double partial verification when evaluating two index tests in relation to a reference test? Statistics in Medicine. 2012;31(11-12):1265-76.

36. Van Geloven N, Broeze KA, Opmeer BC, Mol BW, Zwinderman AH. Correction: How to deal with double partial verification when evaluating two index tests in relation to a reference test? Statistics in Medicine. 2012;31(28):3787-8.

37. Xue X, Kim MY, Castle PE, Strickler HD. A new method to address verification bias in studies of clinical screening tests: Cervical cancer screening assays as an example. Journal of Clinical Epidemiology. 2014;67(3):343-53. doi: 10.1016/j.jclinepi.2013.09.013.

38. Barnhart HX, Kosinki AS. Evaluating medical diagnostic tests at the subunit level in the presence of verification bias. Statistics in Medicine. 2003;22(13):2161-76. doi: 10.1002/sim.1436.

39. Lin CY, Barnhart HX, Kosinski AS. The weighted generalized estimating equations approach for the evaluation of medical diagnostic test at subunit level. Biometrical Journal. 2006;48(5):758-71. doi: 10.1002/bimj.200510199.

40. Dorfman DD, Alf Jr E. Maximum-likelihood estimation of parameters of signal-detection theory and determination of confidence intervals—rating-method data. Journal of mathematical psychology. 1969;6(3):487-96.

41. Bamber D. The area above the ordinal dominance graph and the area below the receiver operating characteristic graph. Journal of mathematical psychology. 1975;12(4):387-415.

42. Gray R, Begg CB, Greenes RA. Construction of receiver operating characteristic curves when disease verification is subject to selection bias. Medical Decision Making. 1984;4(2):151-64.

43. Zhou XH. A nonparametric maximum likelihood estimator for the receiver operating characteristic curve area in the presence of verification bias. Biometrics. 1996;52(1):299-305. doi: 10.2307/2533165.

44. Zhou XH, Rodenberg CA. Estimating an ROC curve in the presence of non-ignorable verification bias. Communications in Statistics - Theory and Methods. 1998;27(3):635-57. doi: 10.1080/03610929808832118.

45. Rodenberg CZ, X. H. ROC curve estimation when covariates affect the verification process. Biometrics. 2000;56(4):1256-62.

46. Hunink MG, Richardson DK, Doubilet PM, Begg CB. Testing for fetal pulmonary maturity: ROC analysis involving covariates, verification bias, and combination testing. Medical Decision Making. 1990;10(3):201-11.

47. Liu DZ, Xiao-Hua. A Model for Adjusting for Nonignorable Verification Bias in Estimation of the ROC Curve and Its Area with Likelihood-Based Approach. Biometrics. 2010;66(4):1119-28. doi: 10.1111/j.1541-0420.2010.01397.x.

48. Fluss RR, Benjamin; Faraggi, David; Rotnitzky, Andrea. Estimation of the ROC Curve under Verification Bias. Biometrical Journal. 2009;51(3):475-90. doi: 10.1002/bimj.200800128.

49. Rotnitzky A, Faraggi D, Schisterman E. Doubly robust estimation of the area under the receiver-operating characteristic curve in the presence of verification bias. Journal of the American Statistical Association. 2006;101(475):1276-88. doi: 10.1198/016214505000001339.

50. He HL, Jeffrey M.; McDermott, Michael P. Direct estimation of the area under the receiver operating characteristic curve in the presence of verification bias. Statistics in Medicine. 2009;28(3):361-76. doi: 10.1002/sim.3388.

51. Gu J, Ghosal S. Bayesian ROC curve estimation under binormality using a rank likelihood. Journal of Statistical Planning and Inference. 2009;139(6):2076-83. doi: <https://doi.org/10.1016/j.jspi.2008.09.014>.

52. Gu J, Ghosal S, Kleiner DE. Bayesian ROC curve estimation under verification bias. Statistics in Medicine. 2014;33(29):5081-96. doi: 10.1002/sim.6297.

53. Ning J, Cheng PE. A comparison study of nonparametric imputation methods. Statistics and Computing. 2012;22(1):273-85. doi: 10.1007/s11222-010-9223-y.

54. Adimari G, Chiogna M. Nearest-neighbor estimation for ROC analysis under verification bias. International Journal of Biostatistics. 2015;11(1):109-24. doi: 10.1515/ijb-2014-0014.

55. Adimari G, Chiogna M. Nonparametric verification bias-corrected inference for the area under the ROC curve of a continuous-scale diagnostic test. Statistics and its Interface. 2017;10(4):629-41. doi: 10.4310/SII.2017.v10.n4.a8.

56. Yu W, Kim JK, Park T. ESTIMATION OF AREA UNDER THE ROC CURVE UNDER NONIGNORABLE VERIFICATION BIAS. Statistica Sinica. 2018;28(4):2149-66.

57. Page JH, Rotnitzky A. Estimation of the disease-specific diagnostic marker distribution under verification bias. Computational Statistics and Data Analysis. 2009;53(3):707-17. doi: 10.1016/j.csda.2008.06.021.

58. Liu DZ, Xiao-Hua. Semiparametric Estimation of the Covariate-Specific ROC Curve in Presence of Ignorable Verification Bias. Biometrics. 2011;67(3):906-16. doi: 10.1111/j.1541-0420.2011.01562.x.

59. Fluss R, Reiser B, Faraggi D. Adjusting ROC curves for covariates in the presence of verification bias. Journal of Statistical Planning and Inference. 2012;142(1):1-11.

60. Liu DZ, Xiao-Hua. Covariate Adjustment in Estimating the Area Under ROC Curve with Partially Missing Gold Standard. Biometrics. 2013;69(1):91-100. doi: 10.1111/biom.12001.

61. Zhou XH, Castelluccio P. Nonparametric analysis for the ROC areas of two diagnostic tests in the presence of nonignorable verification bias. Journal of Statistical Planning and Inference. 2003;115(1):193-213. doi: 10.1016/S0378-3758(02)00146-5.

62. Yu BZ, Chuan. Assessing the accuracy of a multiphase diagnosis procedure for dementia. Journal of the Royal Statistical Society: Series C (Applied Statistics). 2012;61(1):67-81. doi: 10.1111/j.1467-9876.2011.00771.x.

63. Chi Y-YZ, Xiao-Hua. Receiver operating characteristic surfaces in the presence of verification bias. Journal of the Royal Statistical Society: Series C (Applied Statistics). 2008;57(1):1-23. doi: 10.1111/j.1467-9876.2007.00597.x.

64. Duc KT, Chiogna M, Adimari G. Bias–corrected methods for estimating the receiver operating characteristic surface of continuous diagnostic tests. Electronic Journal of Statistics. 2016;10(2):3063-113. doi: 10.1214/16-EJS1202.

65. Duc KT, Chiogna M, Adimari G. Nonparametric Estimation of ROC Surfaces Under Verification Bias. 2016.

66. To Duc K. bcROCsurface: An R package for correcting verification bias in estimation of the ROC surface and its volume for continuous diagnostic tests. BMC Bioinformatics. 2017;18(1). doi: 10.1186/s12859-017-1914-3.

67. Zhang Y, Alonzo TA, for the Alzheimer's Disease Neuroimaging I. Inverse probability weighting estimation of the volume under the ROC surface in the presence of verification bias. Biometrical Journal. 2016;58(6):1338-56. doi: 10.1002/bimj.201500225.

68. Zhang Y, Alonzo TA, for the Alzheimer's Disease Neuroimaging I. Estimation of the volume under the receiver-operating characteristic surface adjusting for non-ignorable verification bias. Statistical Methods in Medical Research. 2018;27(3):715-39. doi: 10.1177/0962280217742541.

69. Zhu R, Ghosal S. Bayesian Semiparametric ROC surface estimation under verification bias. Computational Statistics and Data Analysis. 2019;133:40-52. doi: 10.1016/j.csda.2018.09.003.

70. To Duc K, Chiogna M, Adimari G, for the Alzheimer's Disease Neuroimaging I. Estimation of the volume under the ROC surface in presence of nonignorable verification bias. Statistical Methods and Applications. 2019. doi: 10.1007/s10260-019-00451-3.

71. Chu HZ, Yijie; Cole, Stephen R.; Ibrahim, Joseph G. On the estimation of disease prevalence by latent class models for screening studies using two screening tests with categorical disease status verified in test positives only. Statistics in Medicine. 2010;29(11):1206-18. doi: 10.1002/sim.3862.

72. Emerson SC, Waikar SS, Fuentes C, Bonventre JV, Betensky RA. Biomarker validation with an imperfect reference: Issues and bounds. Statistical Methods in Medical Research. 2018;27(10):2933-45. doi: 10.1177/0962280216689806.

73. Hahn AL, Marc; Landt, Olfert; Schwarz, Norbert Georg; Frickmann, Hagen. Comparison of one commercial and two in-house TaqMan multiplex real-time PCR assays for detection of enteropathogenic, enterotoxigenic and enteroaggregative Escherichia coli. Tropical Medicine & International Health. 2017;22(11):1371-6. doi: 10.1111/tmi.12976.

74. Matos RN, T. F.; Braga, M. M.; Siqueira, W. L.; Duarte, D. A.; Mendes, F. M. Clinical performance of two fluorescence-based methods in detecting occlusal caries lesions in primary teeth. Caries Research. 2011;45(3):294-302. doi: 10.1159/000328673.

75. Mathews WC, Cachay ER, Caperna J, Sitapati A, Cosman B, Abramson I. Estimating the accuracy of anal cytology in the presence of an imperfect reference standard. PLoS ONE. 2010;5(8). doi: 10.1371/journal.pone.0012284.

76. Gart JJ, Buck AA. COMPARISON OF A SCREENING TEST AND A REFERENCE TEST IN EPIDEMIOLOGIC STUDIES .2. A PROBABILISTIC MODEL FOR COMPARISON OF DIAGNOSTIC TESTS. American Journal of Epidemiology. 1966;83(3):593-&. doi: 10.1093/oxfordjournals.aje.a120610. PubMed PMID: WOS:A19667894500018.

77. Brenner H. Correcting for exposure misclassification using an alloyed gold standard. Epidemiology. 1996;7(4):406-10.

78. Staquet M, Rozencweig M, Lee YJ, Muggia FM. Methodology for the assessment of new dichotomous diagnostic tests. Journal of Chronic Diseases. 1981;34(12):599-610. doi: 10.1016/0021-9681(81)90059-X.

79. Albert PS. Estimating diagnostic accuracy of multiple binary tests with an imperfect reference standard. Statistics in Medicine. 2009;28(5):780-97. doi: 10.1002/sim.3514. PubMed PMID: WOS:000263583500004.

80. Juhl DV, A.; Luhm, J.; Ziemann, M.; Hennig, H.; Görg, S. Comparison of the two fully automated anti-HCMV IgG assays: Abbott Architect CMV IgG assay and Biotest anti-HCMV recombinant IgG ELISA. Transfusion Medicine. 2013;23(3):187-94. doi: 10.1111/tme.12036.

81. Hawkins DMG, J. A.; Stephenson, B. Some issues in resolution of diagnostic tests using an imperfect gold standard. Statistics in Medicine. 2001;20(13):1987-2001. doi: 10.1002/sim.819.

82. Van Dyck E, Buvé A, Weiss HA, Glynn JR, Brown DWG, De Deken B, et al. Performance of commercially available enzyme immunoassays for detection of antibodies against herpes simplex virus type 2 in African populations. Journal of Clinical Microbiology. 2004;42(7):2961-5. doi: 10.1128/JCM.42.7.2961-2965.2004.

83. Rostami MNR, B. H.; Aghsaghloo, F.; Nazari, R. Comparison of clinical performance of antigen based-enzyme immunoassay (EIA) and major outer membrane protein (MOMP)-PCR for detection of genital Chlamydia trachomatis infection. International Journal of Reproductive Biomedicine. 2016;14(6):411-20. PubMed PMID: WOS:000388374300007.

84. Spada EP, Daniela; Baggiani, Luciana; Bagnagatti De Giorgi, Giada; Perego, Roberta; Ferro, Elisabetta. Evaluation of an immunochromatographic test for feline AB system blood typing. Journal of Veterinary Emergency and Critical Care. 2016;26(1):137-41. doi: 10.1111/vec.12360.

85. Brocchi E, Bergmann IE, Dekker A, Paton DJ, Sammin DJ, Greiner M, et al. Comparative evaluation of six ELISAs for the detection of antibodies to the non-structural proteins of foot-and-mouth disease virus. Vaccine. 2006;24(47):6966-79. doi: <https://doi.org/10.1016/j.vaccine.2006.04.050>.

86. Hadgu A. The discrepancy in discrepant analysis. Lancet. 1996;348(9027):592-3. doi: 10.1016/s0140-6736(96)05122-7. PubMed PMID: WOS:A1996VF18600016.

87. Hadgu A, Dendukuri N, Hilden J. Evaluation of nucleic acid amplification tests in the absence of a perfect gold-standard test: a review of the statistical and epidemiologic issues. Epidemiology. 2005:604-12.

88. Schiller IvS, M.; Hadgu, A.; Libman, M.; Reitsma, J. B.; Dendukuri, N. Bias due to composite reference standards in diagnostic accuracy studies. Statistics in Medicine. 2016;35(9):1454-70.

89. Hui SL, Zhou XH. Evaluation of diagnostic tests without gold standards. Statistical Methods in Medical Research. 1998;7(4):354-70.

90. Walter SD, Irwig LM. Estimation of test error rates, disease prevalence and relative risk from misclassified data: a review. Journal of Clinical Epidemiology. 1988;41(9):923-37. doi: 10.1016/0895-4356(88)90110-2.

91. Williams GJM, Petra; Kerr, Marianne; Fitzgerald, Dominic A.; Isaacs, David; Codarini, Miriam; McCaskill, Mary; Prelog, Kristina; Craig, Jonathan C. Variability and accuracy in interpretation of consolidation on chest radiography for diagnosing pneumonia in children under 5 years of age. Pediatric Pulmonology. 2013;48(12):1195-200. doi: 10.1002/ppul.22806.

92. See CWA, W.; Melese, M.; Zhou, Z.; Porco, T. C.; Shiboski, S.; Gaynor, B. D.; Eng, J.; Keenan, J. D.; Lietman, T. M. How reliable are tests for trachoma? - A latent class approach. Investigative Ophthalmology and Visual Science. 2011;52(9):6133-7.

93. Nérette P, Dohoo I, Hammell L. Estimation of specificity and sensitivity of three diagnostic tests for infectious salmon anaemia virus in the absence of a gold standard. Journal of Fish Diseases. 2005;28(2):89-99. doi: 10.1111/j.1365-2761.2005.00612.x.

94. Pak SIK, D. Evaluation of diagnostic performance of a polymerase chain reaction for detection of canine Dirofilaria immitis. Journal of Veterinary Clinics. 2007;24(2):77-81.

95. Asselineau J, Paye A, Bessède E, Perez P, Proust-Lima C. Different latent class models were used and evaluated for assessing the accuracy of campylobacter diagnostic tests: Overcoming imperfect reference standards? Epidemiology and Infection. 2018;146(12):1556-64. doi: 10.1017/S0950268818001723.

96. Jokinen J, Snellman M, Palmu AA, Saukkoriipi A, Verlant V, Pascal T, et al. Testing Pneumonia Vaccines in the Elderly: Determining a Case Definition for Pneumococcal Pneumonia in the Absence of a Gold Standard. American Journal of Epidemiology. 2018;187(6):1295-302. doi: 10.1093/aje/kwx373.

97. Santos FLN, Campos ACP, Amorim LDAF, Silva ED, Zanchin NIT, Celedon PAF, et al. Highly accurate chimeric proteins for the serological diagnosis of chronic chagas disease: A latent class analysis. American Journal of Tropical Medicine and Hygiene. 2018;99(5):1174-9. doi: 10.4269/ajtmh.17-0727.

98. Kang L, Carter R, Darcy K, Kauderer J, Liao S-Y. A fast Monte Carlo EM algorithm for estimation in latent class model analysis with an application to assess diagnostic accuracy for cervical neoplasia in women with AGC. Journal of applied statistics. 2013;40(12):2699.

99. Hagenaars JA. Latent structure models with direct effects between indicators: local dependence models. Sociological Methods & Research. 1988;16(3):379-405.

100. Uebersax JS. Probit latent class analysis with dichotomous or ordered category measures: Conditional independence/dependence models. Applied Psychological Measurement. 1999;23(4):283-97.

101. Xu HB, Michael A.; Craig, Bruce A. Evaluating accuracy of diagnostic tests with intermediate results in the absence of a gold standard. Statistics in Medicine. 2013;32(15):2571-84. doi: 10.1002/sim.5695.

102. Mamtani M, Jawahirani A, Das K, Rughwani V, Kulkarni H. Bias-corrected diagnostic performance of the naked eye single tube red cell osmotic fragility test (NESTROFT): An effective screening tool for β-thalassemia. Hematology. 2006;11(4):277-86. doi: 10.1080/10245330600915875.

103. Sobotzki CR, M.; Kennerknecht, N.; Hulsse, C.; Littmann, M.; White, A.; Von Kries, R.; Von Kotnig, C. H. W. Latent class analysis of diagnostic tests for adenovirus, Bordetella pertussis and influenza virus infections in German adults with longer lasting coughs. Epidemiology and Infection. 2016;144(4):840-6. doi: 10.1017/s0950268815002149. PubMed PMID: WOS:000369712100021.

104. Poynard TDL, V.; Zarski, J. P.; Stanciu, C.; Munteanu, M.; Vergniol, J.; France, J.; Trifan, A.; Le Naour, G.; Vaillant, J. C.; Ratziu, V.; Charlotte, F. Relative performances of FibroTest, Fibroscan, and biopsy for the assessment of the stage of liver fibrosis in patients with chronic hepatitis C: A step toward the truth in the absence of a gold standard. Journal of Hepatology. 2012;56(3):541-8. doi: 10.1016/j.jhep.2011.08.007.

105. De La Rosa GDV, M. L.; Arango, C. M.; Gomez, C. I.; Garcia, A.; Ospina, S.; Osorno, S.; Henao, A.; Jaimes, F. A. Toward an operative diagnosis in sepsis: A latent class approach. BMC Infectious Diseases. 2008;8 (no pagination)(18).

106. Albert PS, McShane LM, Shih JH, Network USNCIBTM. Latent class modeling approaches for assessing diagnostic error without a gold standard: with applications to p53 immunohistochemical assays in bladder tumors. Biometrics. 2001;57(2):610-9.

107. Zhang BC, Z.; Albert, P. S. Estimating Diagnostic Accuracy of Raters Without a Gold Standard by Exploiting a Group of Experts. Biometrics. 2012;68(4):1294-302.

108. Xie YC, Zhen; Albert, Paul S. A crossed random effects modeling approach for estimating diagnostic accuracy from ordinal ratings without a gold standard. Statistics in Medicine. 2013;32(20):3472-85. doi: 10.1002/sim.5784.

109. Liu WZ, B.; Zhang, Z. W.; Chen, B. J.; Zhou, X. H. A pseudo-likelihood approach for estimating diagnostic accuracy of multiple binary medical tests. Computational Statistics & Data Analysis. 2015;84:85-98. doi: 10.1016/j.csda.2014.11.006. PubMed PMID: WOS:000348263200007.

110. Xue X, Oktay M, Goswami S, Kim MY. A method to compare the performance of two molecular diagnostic tools in the absence of a gold standard. Statistical Methods in Medical Research. 2019;28(2):419-31. doi: 10.1177/0962280217726804.

111. Joseph LG, T. W.; Coupal, L. Bayesian estimation of disease prevalence and the parameters of diagnostic tests in the absence of a gold standard. American Journal of Epidemiology. 1995;141(3):263-72.

112. Dendukuri NJ, L. Bayesian approaches to modeling the conditional dependence between multiple diagnostic tests. Biometrics. 2001;57(1):158-67. doi: 10.1111/j.0006-341X.2001.00158.x.

113. Johnson WO, Gastwirth JL, Pearson LM. Screening without a "gold standard": The Hui-Walter paradigm revisited. American Journal of Epidemiology. 2001;153(9):921-4. doi: 10.1093/aje/153.9.921.

114. Georgiadis MP, Johnson WO, Gardner IA, Singh R. Correlation‐adjusted estimation of sensitivity and specificity of two diagnostic tests. Journal of the Royal Statistical Society: Series C (Applied Statistics). 2003;52(1):63-76.

115. Nérette P, Stryhn H, Dohoo I, Hammell L. Using pseudogold standards and latent-class analysis in combination to evaluate the accuracy of three diagnostic tests. Preventive veterinary medicine. 2008;85(3-4):207-25.

116. Dendukuri N, Hadgu A, Wang L. Modeling conditional dependence between diagnostic tests: a multiple latent variable model. Statistics in medicine. 2009;28(3):441-61.

117. Dendukuri NW, L.; Hadgu, A. Evaluating diagnostic tests for Chlamydia trachomatis in the absence of a gold standard: A comparison of three statistical methods. Statistics in Biopharmaceutical Research. 2011;3(2):385-97.

118. Martinez EZL-N, F.; Derchain, S. F. M.; Achcar, J. A.; Gontijo, R. C.; Sarian, L. O. Z.; Syrjänen, K. J. Bayesian estimation of performance measures of cervical cancer screening tests in the presence of covariates and absence of a gold standard. Cancer Informatics. 2008;6:33-46.

119. Zhang JC, Kathryn; McLinden, James H.; Stapleton, Jack T. Bayesian analysis and classification of two enzyme-linked immunosorbent assay tests without a gold standard. Statistics in Medicine. 2013;32(23):4102-17. doi: 10.1002/sim.5816.

120. Dufour SD, J.; Dubuc, J.; Dendukuri, N.; Hassan, S.; Buczinski, S. Bayesian estimation of sensitivity and specificity of a milk pregnancy-associated glycoprotein-based ELISA and of transrectal ultrasonographic exam for diagnosis of pregnancy at 28-45 days following breeding in dairy cows. Preventive Veterinary Medicine. 2017;140:122-33.

121. García Barrado L, Coart E, Burzykowski T. Estimation of diagnostic accuracy of a combination of continuous biomarkers allowing for conditional dependence between the biomarkers and the imperfect reference-test. Biometrics. 2017;73(2):646-55. doi: 10.1111/biom.12583.

122. Lu Y, Dendukuri N, Schiller I, Joseph L. A Bayesian approach to simultaneously adjusting for verification and reference standard bias in diagnostic test studies. Statistics in Medicine. 2010;29(24):2532-43. doi: 10.1002/sim.4018.

123. Habib IS, I.; Uyttendaele, M.; De Zutter, L.; Berkvens, D. A Bayesian modelling framework to estimate Campylobacter prevalence and culture methods sensitivity: application to a chicken meat survey in Belgium. Journal of Applied Microbiology. 2008;105(6):2002-8. doi: 10.1111/j.1365-2672.2008.03902.x.

124. Vidal EM, A.; Bertolini, E.; Cambra, M. Estimation of the accuracy of two diagnostic methods for the detection of Plum pox virus in nursery blocks by latent class models. Plant Pathology. 2012;61(2):413-22. doi: 10.1111/j.1365-3059.2011.02505.x.

125. Aly SS, Anderson RJ, Whitlock RH, Adaska JM. Sensitivity and specificity of two enzyme-linked immunosorbent assays and a quantitative real-time polymerase chain reaction for bovine paratuberculosis testing of a large dairy herd. International Journal of Applied Research in Veterinary Medicine. 2014;12(1):1-7.

126. Rahman AKMA, Saegerman C, Berkvens D, Fretin D, Gani MO, Ershaduzzaman M, et al. Bayesian estimation of true prevalence, sensitivity and specificity of indirect ELISA, Rose Bengal Test and Slow Agglutination Test for the diagnosis of brucellosis in sheep and goats in Bangladesh. Preventive Veterinary Medicine. 2013;110(2):242-52.

127. Nerette P, Dohoo I, Hammell L. Estimation of specificity and sensitivity of three diagnostic tests for infectious salmon anaemia virus in the absence of a gold standard. Journal of Fish Diseases. 2005;28(2):89-99. doi: 10.1111/j.1365-2761.2005.00612.x. PubMed PMID: WOS:000226964500004.

128. Nérette P, Hammell L, Dohoo I, Gardner I. Evaluation of testing strategies for infectious salmon anaemia and implications for surveillance and control programs. Aquaculture. 2008;280(1-4):53-9.

129. Sidibe CAKG, V.; Thiaucourt, F.; Niang, M.; Lesnoff, M.; Roger, F. Performance evaluation of two serological tests for contagious bovine pleuropneumonia (CBPP) detection in an enzootic area using a Bayesian framework. Tropical Animal Health and Production. 2012;44(6):1233-8. doi: 10.1007/s11250-011-0063-3. PubMed PMID: WOS:000305679400015.

130. Speybroeck NP, N.; Claes, F.; van Hong, N.; Torres, K.; Mao, S.; van den Eede, P.; Thinh, T. T.; Gamboa, D.; Sochantha, T.; Thang, N. D.; Coosemans, M.; Buscher, P.; D'Alessandro, U.; Berkvens, D.; Erhart, A. True versus apparent Malaria infection prevalence: The contribution of a Bayesian approach. PLoS ONE. 2011;6 (2) (no pagination)(e16705).

131. Praet NV, Jaco J.; Mwape, Kabemba E.; Phiri, Isaac K.; Muma, John B.; Zulu, Gideon; van Lieshout, Lisette; Rodriguez-Hidalgo, Richar; Benitez-Ortiz, Washington; Dorny, Pierre; Gabriël, Sarah. Bayesian modelling to estimate the test characteristics of coprology, coproantigen ELISA and a novel real-time PCR for the diagnosis of taeniasis. Tropical Medicine & International Health. 2013;18(5):608-14. doi: 10.1111/tmi.12089.

132. Jacobson MW, P.; Nordengrahn, A.; Merza, M.; Emanuelson, U. Evaluation of a blocking ELISA for the detection of antibodies against Lawsonia intracellularis in pig sera. Acta veterinaria Scandinavica. 2011;53:23.

133. Dufour SD, J.; Dubuc, J.; Dendukuri, N.; Hassan, S.; Buczinski, S. Bayesian estimation of sensitivity and specificity of a milk pregnancy-associated glycoprotein-based ELISA and of transrectal ultrasonographic exam for diagnosis of pregnancy at 28–45 days following breeding in dairy cows. Preventive Veterinary Medicine. 2017;140:122-33. doi: 10.1016/j.prevetmed.2017.03.008.

134. Bermingham MLH, I. G.; Glass, E. J.; Woolliams, J. A.; Bronsvoort, B. M. D. C.; McBride, S. H.; Skuce, R. A.; Allen, A. R.; McDowell, S. W. J.; Bishop, S. C. Hui and Walter's latent-class model extended to estimate diagnostic test properties from surveillance data: A latent model for latent data. Scientific Reports. 2015;5. doi: 10.1038/srep11861.

135. Busch ELD, P. K.; Chu, H. T.; Richardson, D. B.; Keku, T. O.; Eberhard, D. A.; Avery, C. L.; Sandler, R. S. Diagnostic accuracy and prediction increment of markers of epithelial-mesenchymal transition to assess cancer cell detachment from primary tumors. Bmc Cancer. 2018;18. doi: 10.1186/s12885-017-3964-3. PubMed PMID: WOS:000422881400003.

136. De Waele V, Berzano M, Berkvens D, Speybroeck N, Lowery C, Mulcahy GM, et al. Age-Stratified Bayesian Analysis To Estimate Sensitivity and Specificity of Four Diagnostic Tests for Detection of Cryptosporidium Oocysts in Neonatal Calves. Journal of Clinical Microbiology. 2011;49(1):76-84. doi: 10.1128/jcm.01424-10. PubMed PMID: WOS:000285787100010.

137. Busch EL, Don PK, Chu H, Richardson DB, Keku TO, Eberhard DA, et al. Diagnostic accuracy and prediction increment of markers of epithelial-mesenchymal transition to assess cancer cell detachment from primary tumors. BMC Cancer. 2018;18(1). doi: 10.1186/s12885-017-3964-3.

138. Falley BN, Stamey JD, Beaujean AA. Bayesian estimation of logistic regression with misclassified covariates and response. Journal of Applied Statistics. 2018;45(10):1756-69. doi: 10.1080/02664763.2017.1391182.

139. Lurier T, Delignette-Muller ML, Rannou B, Strube C, Arcangioli MA, Bourgoin G. Diagnosis of bovine dictyocaulosis by bronchoalveolar lavage technique: A comparative study using a Bayesian approach. Preventive Veterinary Medicine. 2018;154:124-31. doi: 10.1016/j.prevetmed.2018.03.017.

140. Yan Q, Karau MJ, Greenwood-Quaintance KE, Mandrekar JN, Osmon DR, Abdel MP, et al. Comparison of diagnostic accuracy of periprosthetic tissue culture in blood culture bottles to that of prosthesis sonication fluid culture for diagnosis of prosthetic joint infection (PJI) by use of Bayesian latent class modeling and IDSA PJI criteria for classification. Journal of Clinical Microbiology. 2018;56(6). doi: 10.1128/JCM.00319-18.

141. Heida A, Van De Vijver E, Van Ravenzwaaij D, Van Biervliet S, Hummel TZ, Yuksel Z, et al. Predicting inflammatory bowel disease in children with abdominal pain and diarrhoea: Calgranulin-C versus calprotectin stool tests. Archives of Disease in Childhood. 2018;103(6):565-71. doi: 10.1136/archdischild-2017-314081.

142. McDonald JL, Hodgson DJ. Prior precision, prior accuracy, and the estimation of disease prevalence using imperfect diagnostic tests. Frontiers in Veterinary Science. 2018;5(MAY). doi: 10.3389/fvets.2018.00083.

143. Branscum AJ, Gardner IA, Johnson WO. Estimation of diagnostic-test sensitivity and specificity through Bayesian modeling. Preventive veterinary medicine. 2005;68(2-4):145-63.

144. Berkvens D, Speybroeck N, Praet N, Adel A, Lesaffre E. Estimating disease prevalence in a Bayesian framework using probabilistic constraints. Epidemiology. 2006;17(2):145-53.

145. Nielsen SS, Grønbæk C, Agger JF, Houe H. Maximum-likelihood estimation of sensitivity and specificity of ELISAs and faecal culture for diagnosis of paratuberculosis. Preventive Veterinary Medicine. 2002;53(3):191-204. doi: <https://doi.org/10.1016/S0167-5877(01)00280-X>.

146. Henkelman RM, Kay I, Bronskill MJ. Receiver operator characteristic (ROC) analysis without truth. Medical Decision Making. 1990;10(1):24-9.

147. Beiden SV, Campbell G, Meier KL, Wagner RF, editors. The problem of ROC analysis without truth: The EM algorithm and the information matrix. Medical Imaging 2000: Image Perception and Performance; 2000: International Society for Optics and Photonics.

148. Zhou X-HC, Pete; Zhou, Chuan. Nonparametric Estimation of ROC Curves in the Absence of a Gold Standard. Biometrics. 2005;61(2):600-9. doi: 10.1111/j.1541-0420.2005.00324.x.

149. Wang Z, Zhou X-H, Wang M. Evaluation of diagnostic accuracy in detecting ordered symptom statuses without a gold standard. Biostatistics. 2011;12(3):567-81. doi: 10.1093/biostatistics/kxq075.

150. Wang ZZ, Xiao-Hua. Random effects models for assessing diagnostic accuracy of traditional Chinese doctors in absence of a gold standard. Statistics in Medicine. 2012;31(7):661-71. doi: 10.1002/sim.4275.

151. Choi YK, Johnson WO, Collins MT, Gardner IA. Bayesian inferences for receiver operating characteristic curves in the absence of a gold standard. Journal of Agricultural, Biological, and Environmental Statistics. 2006;11(2):210-29. doi: 10.1198/108571106X110883.

152. Wang CT, B. W.; Grohn, Y. T.; Nielsen, S. S. Nonparametric estimation of ROC curves based on Bayesian models when the true disease state is unknown. Journal of Agricultural Biological and Environmental Statistics. 2007;12(1):128-46. doi: 10.1198/108571107x178095. PubMed PMID: WOS:000244815500008.

153. Branscum AJJ, Wesley O.; Hanson, Timothy E.; Gardner, Ian A. Bayesian semiparametric ROC curve estimation and disease diagnosis. Statistics in Medicine. 2008;27(13):2474-96. doi: 10.1002/sim.3250.

154. Jafarzadeh SRJ, Wesley O.; Utts, Jessica M.; Gardner, Ian A. Bayesian estimation of the receiver operating characteristic curve for a diagnostic test with a limit of detection in the absence of a gold standard. Statistics in Medicine. 2010;29(20):2090-106. doi: 10.1002/sim.3975.

155. Hall P, Zhou X-H. Nonparametric estimation of component distributions in a multivariate mixture. The annals of statistics. 2003;31(1):201-24.

156. Erkanli AS, Minje; Jane Costello, E.; Angold, Adrian. Bayesian semi-parametric ROC analysis. Statistics in Medicine. 2006;25(22):3905-28. doi: 10.1002/sim.2496.

157. Tang S, Hemyari P, Canchola JA, Duncan J. Dual composite reference standards (dCRS) in molecular diagnostic research: A new approach to reduce bias in the presence of Imperfect reference. Journal of Biopharmaceutical Statistics. 2018;28(5):951-65. doi: 10.1080/10543406.2018.1428613.

158. Saugar JMM, F. J.; Martin-Rabadan, P.; Fernandez-Soto, P.; Ortega, S.; Garate, T.; Rodriguez, E. Application of real-time PCR for the detection of Strongyloides spp. in clinical samples in a reference center in Spain. Acta Tropica. 2015;142:20-5.

159. Peterson LRY, S. A.; Davis, T. E.; Wang, Z. X.; Duncan, J.; Noutsios, C.; Liesenfeld, O.; Osiecki, J. C.; Lewinski, M. A. Evaluation of the cobas cdiff test for detection of toxigenic clostridium difficile in stool samples. Journal of Clinical Microbiology. 2017;55(12):3426-36. doi: 10.1128/JCM.01135-17.

160. Ziswiler HR, Reichenbach S, Vögelin E, Bachmann LM, Villiger PM, Jüni P. Diagnostic value of sonography in patients with suspected carpal tunnel syndrome: A prospective study. Arthritis and Rheumatism. 2005;52(1):304-11. doi: 10.1002/art.20723.

161. Fiebrich HBB, A. H.; Kerstens, M. N.; Pijl, M. E. J.; Kema, I. P.; De Jong, J. R.; Jager, P. L.; Elsinga, P. H.; Dierckx, R. A. J. O.; Van Der Wal, J. E.; Sluiter, W. J.; De Vries, E. G. E.; Links, T. P. 6-[F-18]fluoro-L-dihydroxyphenylalanine positron emission tomography is superior to conventional imaging with123I-metaiodobenzylguanidine scintigraphy, computer tomography, and magnetic resonance imaging in localizing tumors causing catecholamine excess. Journal of Clinical Endocrinology and Metabolism. 2009;94(10):3922-30. doi: 10.1210/jc.2009-1054.

162. Paine SK, Basu A, Choudhury RG, Bhattacharya B, Chatterjee S, Bhattacharya C. Multiplex PCR from Menstrual Blood: A Non-Invasive Cost-Effective Approach to Reduce Diagnostic Dilemma for Genital Tuberculosis. Molecular Diagnosis and Therapy. 2018;22(3):391-6. doi: 10.1007/s40291-018-0322-3.

163. Naaktgeboren CA, Bertens LC, van Smeden M, de Groot JA, Moons KG, Reitsma JB. Value of composite reference standards in diagnostic research. Bmj. 2013;347:f5605.

164. Bertens LC, Broekhuizen BD, Naaktgeboren CA, Rutten FH, Hoes AW, van Mourik Y, et al. Use of expert panels to define the reference standard in diagnostic research: a systematic review of published methods and reporting. PLoS medicine. 2013;10(10):e1001531.

165. Fleiss JL, Cohen J, Everitt BS. Large sample standard errors of kappa and weighted kappa. Psychological bulletin. 1969;72(5):323.

166. Feuer EJ, Kessler LG. Test statistic and sample size for a two-sample McNemar test. Biometrics. 1989:629-36.

167. Hsia ECS, Neil; Cush, John J.; Chaisson, Richard E.; Matteson, Eric L.; Xu, Stephen; Beutler, Anna; Doyle, Mittie K.; Hsu, Benjamin; Rahman, Mahboob U. Interferon-γ release assay versus tuberculin skin test prior to treatment with golimumab, a human anti-tumor necrosis factor antibody, in patients with rheumatoid arthritis, psoriatic arthritis, or ankylosing spondylitis. Arthritis & Rheumatism. 2012;64(7):2068-77. doi: 10.1002/art.34382. PubMed PMID: 104469597. Language: English. Entry Date: 20120717. Revision Date: 20150711. Publication Type: Journal Article.

168. von Heymann W, Moll H, Rauch G. Study on sacroiliac joint diagnostics: Reliability of functional and pain provocation tests. Manuelle Medizin. 2018;56(3):239-48. doi: 10.1007/s00337-018-0405-6.

169. Zaki R, Bulgiba A, Ismail R, Ismail NA. Statistical methods used to test for agreement of medical instruments measuring continuous variables in method comparison studies: a systematic review. PloS one. 2012;7(5):e37908.

170. Elliott DG, Applegate LJ, Murray AL, Purcell MK, McKibben CL. Bench-top validation testing of selected immunological and molecular Renibacterium salmoninarum diagnostic assays by comparison with quantitative bacteriological culture. Journal of Fish Diseases. 2013;36(9):779-809. doi: 10.1111/jfd.12079.

171. Booi ANM, Jerome; Norton, H. James; Anderson, William E.; Ellis, Amy C. Validation of a Screening Tool to Identify Undernutrition in Ambulatory Patients With Liver Cirrhosis. Nutrition in Clinical Practice. 2015;30(5):683-9. doi: 10.1177/0884533615587537.

172. Gorman SLR, S.; Melnick, M. E.; Abrams, G. M.; Byl, N. N. Development and validation of the function in sitting test in adults with acute stroke. Journal of Neurologic Physical Therapy. 2010;34(3):150-60. doi: 10.1097/NPT.0b013e3181f0065f. PubMed PMID: 105109978. Language: English. Entry Date: 20101004. Revision Date: 20150818. Publication Type: Journal Article.

173. Keezer MRP, Amélie; Stechysin, Barbara; Veilleux, Martin; Jetté, Nathalie; Wolfson, Christina. The diagnostic test accuracy of a screening questionnaire and algorithm in the identification of adults with epilepsy. Epilepsia. 2014;55(11):1763-71. doi: 10.1111/epi.12805.

174. Itza F, Zarza D, Salinas J, Teba F, Ximenez C. Turn-amplitude analysis as a diagnostic test for myofascial syndrome in patients with chronic pelvic pain. Pain Research and Management. 2015;20(2):96-100.
